# A CD10‐OGP Membrane Peptolytic Signaling Axis in Fibroblasts Regulates Lipid Metabolism of Cancer Stem Cells via SCD1

**DOI:** 10.1002/advs.202101848

**Published:** 2021-08-07

**Authors:** Shubin Yu, Yiwen Lu, An Su, Jianing Chen, Jiang Li, Boxuan Zhou, Xinwei Liu, Qidong Xia, Yihong Li, Jiaqian Li, Min Huang, Yingying Ye, Qiyi Zhao, Sushi Jiang, Xiaoqing Yan, Xiaojuan Wang, Can Di, Jiayao Pan, Shicheng Su

**Affiliations:** ^1^ Guangdong Provincial Key Laboratory of Malignant Tumor Epigenetics and Gene Regulation Medical Research Center Sun Yat‐Sen Memorial Hospital Sun Yat‐Sen University Guangzhou 510120 China; ^2^ Breast Tumor Center Sun Yat‐Sen Memorial Hospital Sun Yat‐Sen University Guangzhou 510120 China; ^3^ Department of Infectious Diseases the Third Affiliated Hospital Sun Yat‐Sen University Guangzhou 510630 China; ^4^ Guangdong Provincial Key Laboratory of Liver Disease Research the Third Affiliated Hospital Sun Yat‐sen University Guangzhou 510630 China; ^5^ Key Laboratory of Tropical Disease Control (Sun Yat‐sen University) Ministry of Education Guangzhou Guangdong 510080 China; ^6^ Department of Immunology Zhongshan School of Medicine Sun Yat‐Sen University Guangzhou 510080 China

**Keywords:** cancer stem cells, CD10, fibroblast subset, lipid metabolism

## Abstract

Carcinoma‐associated fibroblasts (CAFs) consist of heterogeneous subpopulations that play a critical role in the dynamics of the tumor microenvironment. The extracellular signals of CAFs have been attributed to the extracellular matrix, cytokines, cell surface checkpoints, and exosomes. In the present study, it is demonstrated that the CD10 transmembrane hydrolase expressed on a subset of CAFs supports tumor stemness and induces chemoresistance. Mechanistically, CD10 degenerates an antitumoral peptide termed osteogenic growth peptide (OGP). OGP restrains the expression of rate‐limiting desaturase SCD1 and inhibits lipid desaturation, which is required for cancer stem cells (CSCs). Targeting CD10 significantly improves the efficacy of chemotherapy in vivo. Clinically, CD10‐OGP signals are associated with the response to neoadjuvant chemotherapy in patients with breast cancer. The collective data suggest that a nexus between the niche and lipid metabolism in CSCs is a promising therapeutic target for breast cancer.

## Graphical abstract

CD10, a transmembrane hydrolase expressed by a subset of carcinoma‐associated fibroblasts, drives tumor progression by degrading an anti‐tumoral peptide, osteogenic growth peptide (OGP). OGP impairs tumor lipid desaturation and consequently suppresses cancer stem cells (CSCs). This study sheds new light on a nexus between the niche and lipid metabolism in CSCs.

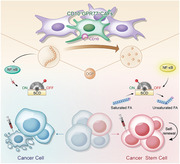

## 1 Introduction

Carcinoma‐associated fibroblasts (CAFs) are activated fibroblasts that are key components of the tumor microenvironment (TME).^[^ ^1^, ^2^ ^]^ CAFs play a crucial role in tumor progression^[^ ^1^, ^3^ ^]^ and have been implicated as promising targets for cancer treatments.^[^ ^4^, ^5^ ^]^ However, phenotypic and functional heterogeneity of CAFs hinders this potential.^[^ ^6^, ^7^ ^]^ Despite the increasing recognition of the functional diversity of CAFs,^[^ ^8^, ^9^ ^]^ definitive surface markers in CAFs for precision therapies are still very limited.^[^ ^10^ ^]^ Dissecting the therapeutic value of CAF subset markers is important to design more effective therapies that leverage knowledge of the deleterious and beneficial roles of CAFs.

Cancer stem cells (CSCs) constitute a small proportion of tumor cells with a strong tumorigenic capacity.^[^ ^11^, ^12^, ^13^, ^14^, ^15^ ^]^ CSCs are responsible for cancer development, disease recurrence, metastasis, and drug resistance in many types of malignancies.^[^ ^16^, ^17^ ^]^ Therapeutic approaches targeting CSCs have been reported to be effective in suppressing tumor development in preclinical studies.^[^ ^18^, ^19^, ^20^, ^21^ ^]^ However, the plasticity of phenotypes and lack of exclusive markers restrict the direct eradication of CSCs. Accumulating evidence indicates that CSCs always occupy specialized niches to maintain their stem cell‐like properties.^[^ ^22^ ^]^ As an important component of CSC niches, CAFs play a critical role in regulating CSC dynamics.^[^ ^23^ ^]^ Therefore, selective targeting of CAFs that dictate the fate of CSCs may be a promising therapeutic strategy.^[^ ^24^, ^25^ ^]^

Previously, we identified a subset of CD10^+^GPR77^+^ CAFs that promote tumor formation and chemoresistance by providing a survival niche for CSCs.^[^ ^25^ ^]^ The induction of CD10^+^GPR77^+^ CAFs is triggered by complement signaling via GPR77. CD10^+^GPR77^+^ CAFs sustain tumor stemness by the secretion of interleukin (IL)‐6 and IL‐8. However, the role of CD10 in CAFs is unknown. CD10 is a transmembrane zinc‐dependent metallopeptidase^[^ ^26^ ^]^ that hydrolyzes multiple bioactive peptides^[^ ^27^ ^]^ and plays a pivotal role in various diseases, including skin inflammation, multiple sclerosis, and several age‐related diseases.^[^ ^28^, ^29^, ^30^ ^]^ However, the function of CD10 in cancer and its underlying mechanisms remain largely unknown. Here, we investigated the function of CD10 in CAFs and explored its underlying mechanisms and therapeutic value.

## 2 Results

### 2.1 CD10 in CAFs Sustains Cancer Stemness and Chemoresistance

**Figure 1:**
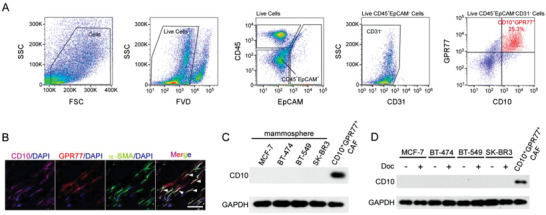
CD10 expression in CAFs. A) Representative flow cytometry plot of gating strategy to identify the CD10^+^GPR77^+^ CAF subset in breast cancer clinical samples. B) Representative images of CD10, GPR77, and *α*‐SMA immunofluorescence staining in human breast cancer sections. White arrows indicate CD10^+^GPR77^+^ CAFs. Scale bar, 50 µm. C) CD10 levels in indicated mammospheres and CD10^+^GPR77^+^ CAFs (*n* = 3). D) CD10 levels in indicated tumor cells treated with docetaxel and CD10^+^GPR77^+^ CAFs (*n* = 3).

Consistent with our previous report,^[^ ^25^ ^]^ flow cytometry analysis of single cell suspensions isolated from human breast cancer (**Figure** **1**A) and immunofluorescence staining of clinical samples (Figure 1B) revealed that CD10 in tumors was predominantly expressed by a subset of GPR77^+^ CAFs negative for the pan‐leukocyte marker CD45, and the epithelial cell adhesion molecule (EpCAM), and the endothelial cell markers CD31. It has been reported that CD10 may also be expressed in CSCs of other tumor types.^[^ ^31^, ^32^ ^]^ However, online transcriptional data showed that the expression of CD10 was absent in breast cancer cell lines (Figure S1A, Supporting Information). In agreement, we found that the CD10 was barely detectable in mammospheres or docetaxel‐treated breast cancer cells (Figure 1C,D). These data suggest that CD10 is predominantly expressed in a fibroblast subset of breast cancer.

**Figure 2:**
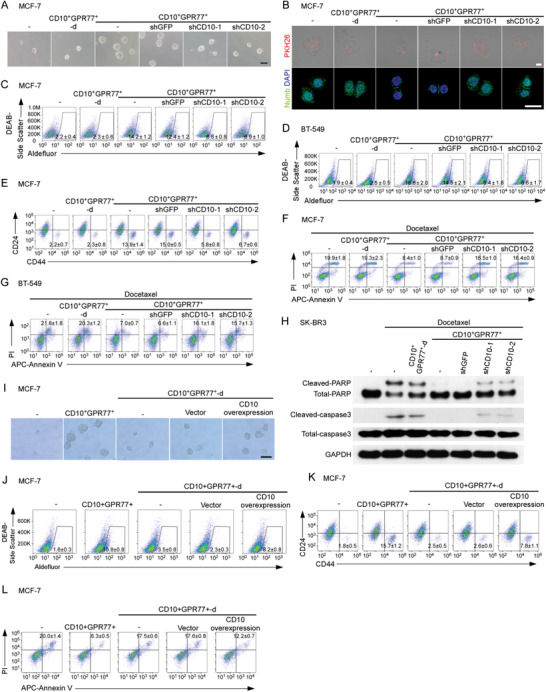
CD10 in CAFs sustains cancer stemness and chemoresistance. A–E) Indicated tumor cell lines were cultured alone (−) or cocultured with CD10^+^GPR77^+^‐depleted CAFs (CD10^+^GPR77^+^‐d) or paired CD10^+^GPR77^+^ CAFs transduced without (−) or with shGFP or shCD10. A) Representative images of mammosphere formation in MCF‐7 cells. Scale bar, 100 µm. B) Representative images of PKH26 and Numb immunofluorescence staining of MCF‐7 cells. Scale bar, 50 µm. C,D) Percentage of ALDH1^+^ cells in C) MCF‐7 and D) BT‐549 cells detected by flow cytometry. Numerical values are presented as percentage (mean ± SEM, *n* = 3). E) Percentage of CD44^high^CD24^low^ cells in MCF‐7 cells cocultured with indicated CAFs was detected by flow cytometry. Numerical values are presented as percentage (mean ± SEM, *n* = 3). F,G) Representative flow cytometry plots for F) MCF‐7 and G) BT‐549 cells treated with docetaxel after being cultured alone (−) or cocultured with the indicated CAFs. The proportion of Annexin V^+^/Propidium iodide^−^ (early apoptosis) and Annexin V^+^/Propidium iodide^+^ (late apoptosis) cells is shown. The numerical values indicate Annexin V^+^ percentage. Data are represented as the mean ± SEM of F) *n* = 4 or G) *n* = 3 independent experiments. H) Representative immunoblots for cleaved/total caspase‐3 and PARP in SK‐BR3 cells treated with docetaxel after being cultured alone (−) or cocultured with indicated CAFs (*n* = 3). I–L) MCF‐7 cells were cocultured with CD10^+^GPR77^+^ CAFs or CD10^+^GPR77^+^‐depleted CAFs transduced with lentiviral empty vectors (vector) or CD10‐expressing vectors (CD10 overexpression). I) Representative images of mammosphere formation. Scale bar, 100 µm. J) Percentage of ALDH1^+^ cells detected by flow cytometry. Numerical values are presented as percentage (mean ± SEM, *n* = 3). K) Pcercentage of CD44^high^CD24^low^ cells detected by flow cytometry. Numerical values are presented as percentage (mean ± SEM, *n* = 3). L) After the coculture, MCF‐7 cells were treated with docetaxel. The proportion of Annexin V^+^/Propidium iodide^−^ and Annexin V^+^/Propidium iodide^+^ was detected by flow cytometry. The numerical values indicate Annexin V^+^ percentage (mean ± SEM, *n* = 3).

To investigate the contribution of CD10 in CAFs to tumor stemness and chemoresistance, we employed a coculture model consisting of breast tumor cells and primary CAFs isolated as previously described.^[^ ^25^ ^]^ CD10 was knocked down in CD10^+^GPR77^+^ primary CAFs isolated from breast cancer tissues of patients (Figure S2A–C, Supporting Information). Breast cancer cells were cocultured with CD10^+^GPR77^+^ CAFs with or without CD10 knockdown or CD10^+^GPR77^+^‐depleted (CD10^+^GPR77^+^‐d) CAFs. After 2 weeks, breast cancer cells cocultured with CD10^+^GPR77^+^ CAFs generated significantly more mammospheres (**Figure** **2**A; Figure S2D, Supporting Information), and demonstrated increased asymmetrical divisions (Figure 2B; Figure S2E, Supporting Information) compared to those cocultured with CD10^+^GPR77^+^‐depleted CAFs. Additionally, the proportions of both ALDH1^+^ and CD44^high^CD24^low^ cells^[^ ^33^ ^]^ were markedly higher in tumor cells cocultured with CD10^+^GPR77^+^ CAFs (Figure 2C–E; Figure S2F–H, Supporting Information). Silencing CD10 expression in CAFs significantly abrogated these effects (Figure 2A–E; Figure S2D–H, Supporting Information), suggesting that CD10 in CAFs plays a crucial role in supporting CSCs. Furthermore, tumor cells cocultured with CAFs were challenged with chemotherapy. CD10^+^GPR77^+^ CAFs, but not CD10^+^GPR77^+^‐depleted CAFs, notably protected tumor cells from chemotherapy‐induced apoptosis (Figure 2F–H; Figure S2I,J, Supporting Information). Again, silencing CD10 expression in CAFs abolished these effects (Figure 2F–H; Figure S2I,J, Supporting Information). When CD10^+^GPR77^+^‐depleted CAFs were transduced with CD10‐expressing lentivirus, forced expression of CD10 significantly enhanced the capacity of CAFs to increase mammosphere formation and ALDH1^+^/CD44^high^ CD24^low^ percentage of cocultured tumor cells (Figure 2I–K; Figure S2K–M, Supporting Information). Additionally, CD10 overexpression also promoted the ability of CD10^+^GPR77^+^‐depleted CAFs to diminish chemotherapy‐induced apoptosis of cocultured tumor cells (Figure 2L; Figure S2N, Supporting Information). Together, these results demonstrate the key role of CD10 in CAFs in sustaining breast cancer stemness and chemoresistance. Previously, we reported that IL‐6 and IL‐8 mediated by GPR77 in CD10^+^GPR77^+^ CAFs contributes to CSC support.^[^ ^25^ ^]^ To investigate whether CD10 mediates the expression of IL‐6, IL‐8, and GPR77 in CAFs, we transduced CD10^+^GPR77^+^ CAFs with CD10 short hairpin RNA (shRNA). CD10 knockdown had no appreciable effect on the levels of IL‐6, IL‐8, or GPR77 (Figure S2O,P, Supporting Information). The collective data suggest that CD10 in CAFs sustains cancer stemness and chemoresistance via a GPR77/IL‐6/IL‐8 independent pathway.

### 2.2 OGP is a Substrate of CD10 in Tumors

**Figure 3:**
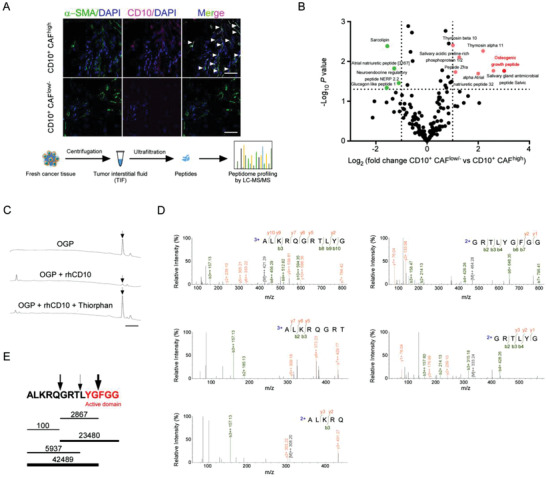
OGP is a substrate of CD10 in tumors. A) Interstitial fluid of breast cancer samples from patients with high or low CD10^+^ CAF infiltration analyzed by HPLC‐MS/MS to identify differentially distributed peptides. Representative immunofluorescent images of samples with high or low CD10^+^ CAFs (upper) and workflow (lower) are shown. White arrows indicate CD10^+^ CAFs. Scale bar, 50 µm. B) The peptide profile identified in A. Red and green dots indicate peptides significantly increased and decreased in samples with low CD10^+^ CAFs compared in those with high CD10^+^ CAFs; fold change > 2, *P* < 0.05. C) OGP was incubated alone (−) (top), or incubated with rhCD10 in the absence (middle) or presence of thiorphan (bottom). The levels of OGP were determined using HPLC. The peak of the OGP is indicated by a black arrow. Scale bar, 2 min. D,E) OGP was incubated with rhCD10 for 1 h. The structure of the hydrolyzed products was determined by HPLC‐MS/MS. D) HPLC‐MS/MS spectrum of hydrolyzed products. The fragments detected by MS/MS are indicated above the peptide sequence for C‐terminal y ions and below the peptide sequence for N‐terminal b ions. E) Peptide fragments in the hydrolyzed products were detected by HPLC‐MS/MS. The horizontal lines indicate the peptide fragments. Thickness indicates the abundance of the fragment. Numbers above the lines indicate MS estimates of the amount of each fragment in arbitrary units. Arrows indicate cleavage sites. The active domain of OGP is marked in red.

CD10 is a cell surface type 2 metalloprotease that can degrade various signaling peptides.^[^ ^27^, ^34^ ^]^ To investigate whether CD10 in CAFs exerts its functions by mediating peptide degradation, high‐performance liquid chromatography‐tandem mass spectrometry (HPLC‐MS/MS) was used to compare peptide profiles in interstitial fluid of breast cancer samples with high or low CD10^+^ CAF infiltration (**Figure** **3**A). The peptidomic analysis revealed that seven peptides were significantly enriched in samples with low CD10^+^ CAF infiltration. Among them, we focused on osteogenic growth peptide (OGP) for three reasons. First, OGP was one of the peptides with the highest fold changes between samples with low CD10^+^ CAF infiltration and those with high CD10^+^ CAF infiltration (Figure 3B). Second, OGP reportedly exhibits antitumor capacity in vitro.^[^ ^35^, ^36^ ^]^ Third, previous studies have shown that OGP regulates the differentiation of mesenchymal stem cells and hematopoietic stem cells.^[^ ^37^, ^38^, ^39^, ^40^ ^]^

To validate whether OGP is a substrate for CD10, HPLC profiles of OGP incubated with or without recombinant human CD10 (rhCD10) were obtained (Figure 3C). The peak height of OGP was significantly reduced after incubation with rhCD10, which could be rescued by thiorphan, a neutral endopeptidase inhibitor (Figure 3C). In addition, the hydrolytic products of OGP in the system were detected by HPLC‐MS/MS. Three cleavage sites on OGP cleaved by rhCD10 were evident. One mapped on the bioactive domain of OGP^[^ ^41^, ^42^ ^]^ (Figure 3D,E). These data indicate that OGP is a substrate of CD10.

### 2.3 CD10 Supports CSCs by Cleaving the C‐Terminal Bioactive Domain in OGP

**Figure 4:**
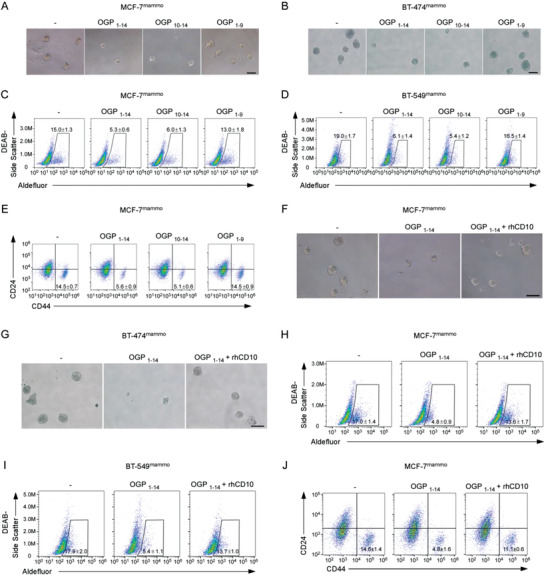
OGP suppresses CSCs via the YGFGG domain that can be cleaved by CD10. A–E) Indicated tumor cell lines after prolonged mammosphere culture were treated with 5× 10^−9^ m OGP_1–14_, OGP_10–14_, or OGP_1–9_, which is approximately equivalent to the OGP concentration of 10% FBS used in conventional cell culture. A,B) Representative images of mammosphere formation in A) MCF‐7^mammo^ cells and B) BT‐474^mammo^ cells. Scale bar, 100 µm. C,D) Percentage of ALDH1^+^ cells in C) MCF‐7^mammo^ cells and D) BT‐549^mammo^ cells (mean ± SEM, *n* = 3). E) Percentage of CD44^high^CD24^low^ cells in MCF‐7^mammo^ cells (mean ± SEM, *n* = 3). F–J) Indicated tumor cell lines after prolonged mammosphere culture were treated with 5 × 10^−9^ m OGP_1–14_ with or without 5 µg mL^−1^ rhCD10. F,G) Representative images of mammosphere formation in F) MCF‐7^mammo^ cells and G) BT‐474^mammo^ cells. Scale bar, 100 µm. H,I) Percentage of ALDH1^+^ cells in H) MCF‐7^mammo^ cells and I) BT‐549^mammo^ cells (mean ± SEM, *n* = 3). J) Percentage of CD44^high^CD24^low^ cells in MCF‐7^mammo^ cells (mean ± SEM, *n* = 3).

OGP plays a crucial role in mediating the differentiation of mesenchymal stem cells and hematopoietic stem cells.^[^ ^37^, ^38^, ^39^, ^40^ ^]^ Whether OGP has any effect on CSCs remains unknown. To assess this, we used tumor cells after prolonged mammosphere culture. These cells are enriched with CSCs as other authors^[^ ^43^ ^]^ and we^[^ ^44^ ^]^ previously described (MCF‐7^mammo^, BT‐474^mammo^, and BT‐549^mammo^). Recombinant full‐length OGP (OGP_1–14_) was added in mammosphere culture. OGP_1–14_ substantially reduced mammosphere formation and the percentage of ALDH1^+^/CD44^high^ CD24^low^ cells in multiple breast cancer cell lines (**Figure** **4**A–J; Figure S3A–J, Supporting Information). The findings indicated that OGP inhibits the stemness of CSCs. It has been reported that the last five amino acids at the C‐terminus of OGP (_YGFGG_) are active domains of OGP.^[^ ^41^, ^45^ ^]^ We investigated whether this domain is indispensable for OGP suppression in CSCs by constructing two deletion versions of OGP. One included the first nine amino acids at the N‐terminus (OGP_1–9_). The other included the last five amino acids at the C‐terminus (OGP_10–14_). Treatment with OGP_10–14_, rather than OGP_1–9_, recapitulated the suppressive effect of wild‐type OGP on CSC traits of tumor cells (Figure 4A–E; Figure S3A–E, Supporting Information). Moreover, concomitant treatment with rhCD10, which can cleave OGP_10–14_ (Figure 3D,E), reversed the effect of wild‐type OGP (Figure 4F–J; Figure S3F–J, Supporting Information). Together, these data suggest that OGP suppresses CSC properties through the YGFGG domain at the C‐terminus that can be cleaved by CD10.

**Figure 5:**
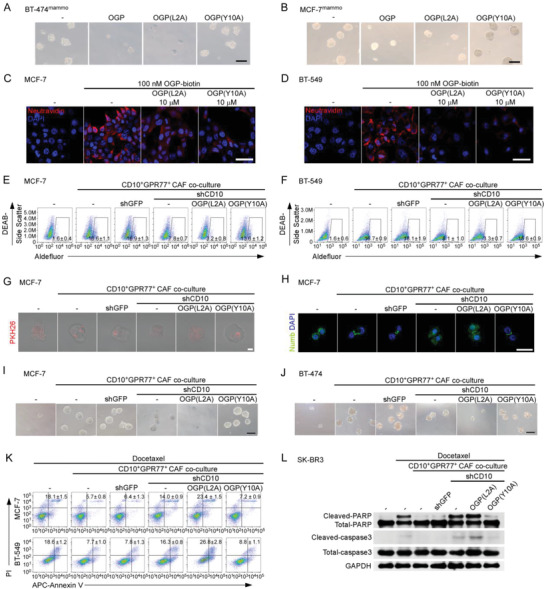
CD10 supports CSCs by cleavage of OGP. A,B) Indicated tumor cell lines after prolonged mammosphere culture were treated with 5 × 10^−9^ m OGP, OGP (L2A), or OGP (Y10A). Representative images of mammosphere formation in A) BT‐474^mammo^ cells and B) MCF‐7^mammo^ cells. Scale bar, 100 µm. C) MCF‐7 and D) BT‐549 cells pretreated with 100 × 10^−9^ m biotinylated OGP, due to an undetectable signal of fluorescence‐labeled peptide at a lower concentration,^[^ ^124^, ^125^, ^126^, ^127^, ^128^, ^129^ ^]^ without (−) or with 10 × 10^−6^ m non‐biotinylated OGP (L2A) or OGP (Y10A) were incubated with neutravidin‐Texas red. The cell surface‐bound biotinylated OGP was evaluated by fluorescence microscopy. Scale bar, 50 µm. E–J) Indicated cell lines were cultured alone (−) or cocultured with the indicated CAFs in the presence of 500 × 10^−9^ m OGP (L2A) or OGP (Y10A). Unlabeled peptides were used at 100 times more than the physiological concentration for the competitive binding assay as previously reported.^[^ ^130^, ^131^ ^]^ E,F) Percentage of ALDH1^+^ cells in E) MCF‐7 and F) BT‐549 cell (mean ± SEM, *n* = 4). G,H) Representative immunofluorescent images of G) PKH26 and H) Numb in MCF‐7 cells. Scale bar, 50 µm. I,J) Representative images of mammosphere formation in I) MCF‐7 and J) BT‐474 cells. Scale bar, 100 µm. K,L) After coculture with (E–J) cancer cells were treated with docetaxel. Apoptosis of tumor cells was determined after 12 h. K) Percentage of apoptotic MCF‐7 and BT‐549 cells evaluated by flow cytometry (mean ± SEM, *n* = 4). L) Apoptosis of SK‐BR3 cells determined by western blotting for cleaved caspase‐3 and PARP (*n* = 3).

Both human and animal serum contain high levels of OGP,^[^ ^46^, ^47^ ^]^ and the amino acid sequences of human and bovine OGP are identical (Figure S4A, Supporting Information). Serum‐containing media inhibit CSC enrichment.^[^ ^48^ ^]^ Given that CD10 can enrich CSCs and degrade OGP, a CSC suppressor, we investigated whether CD10 supports CSCs by inducing OGP cleavage in serum. The OGP receptor remains unidentified.^[^ ^49^ ^]^ Moreover, no effective neutralizing antibodies against OGP are available. Therefore, we employed a competitive binding assay using peptides with point mutations.^[^ ^50^ ^]^ Tyr^10^ in the bioactive domain plays a principal role in the bioactivity of OGP.^[^ ^49^, ^51^ ^]^ Therefore, we constructed two OGP mutants, including OGP (Y10A) with a Tyr^10^ to Ala substitution and OGP (L2A), which harbors a Leu^2^ to Ala substitution in a region not in the bioactive domain. Indeed, OGP (L2A), rather than OGP (Y10A), preserved the inhibitory effect of OGP on mammosphere formation (**Figure** **5**A,B; Figure S4B, Supporting Information). To investigate the capacity of these mutants to compete with wild‐type OGP on the cell surface, we incubated tumor cells in biotin‐conjugated OGP together with OGP (L2A) or OGP (Y10A) at a 100‐fold concentration. OGP binding was detected using fluorescence‐labeled neutravidin and evaluated by fluorescence microscopy and flow cytometry. We observed that excessive amounts of both OGP (L2A) and OGP (Y10A) significantly reduced the binding of wild‐type OGP to the cell surface (Figure 5C,D; Figure S4C,D, Supporting Information).

Consistent with previous reports,^[^ ^46^ ^]^ we found that the concentration of total OGP in human and bovine serum was 14.1 ± 2.9 and 27.8 ± 0.9 × 10^−9^ m, respectively (Figure S4E,F, Supporting Information). Therefore, to investigate the effect of CD10 in OGP‐free conditions, tumor cells and CAFs were cocultured in a culture medium lacking both serum and bovine serum albumin.^[^ ^52^, ^53^, ^54^ ^]^ No appreciable difference was observed in mammosphere formation and chemotherapy‐induced apoptosis between tumor cells cultured alone and those cocultured with CD10^+^GPR77^+^‐depleted CAFs with CD10 forced expression (Figure S4G,H, Supporting Information). Furthermore, the addition of OGP substantially reduced mammosphere formation and increased chemotherapy‐induced apoptosis of MCF‐7^mammo^ cells in serum‐ and BSA‐free medium, whereas addition of rhCD10 abolished these effects (Figure S4I,J, Supporting Information). To explore whether CD10 maintains CSCs by degrading the active domain of OGP, 500 × 10^−9^ m OGP (L2A) and OGP (Y10A), which was about 100 times more than the physiological concentration for the competitive binding assay, were added to the cocultured tumor cells and CD10^+^GPR77^+^ CAFs. Silencing CD10 in CD10^+^GPR77^+^ CAFs markedly reduced the ALDH1^+^ fractions of cocultured tumor cells (Figure 5E,F; Figure S4K,L, Supporting Information). Interestingly, the addition of OGP (L2A) further reduced the proportion of ALDH1^+^ tumor cells cocultured with CD10‐knockdown CAFs. In sharp contrast, the addition of OGP (Y10A) rescued the inhibitory effects of CD10 knockdown on ALDH1^+^ percentage of tumor cells coincubated with CD10^+^GPR77^+^ CAFs. In agreement with these findings, similar rescue effects of OGP (Y10A) were observed for asymmetric division (Figure 5G,H; Figure S4M, Supporting Information), mammosphere formation (Figure 5I,J; Figure S4N,O, Supporting Information), and chemosensitivity (Figure 5K,L; Figure S4P, Supporting Information) in tumor cells cocultured with CD10^+^GPR77^+^ CAFs in which CD10 was silenced. The collective data indicate that CD10 sustains tumor stemness by cleaving the active domain of OGP.

### 2.4 OGP Suppresses CSCs by Inhibiting Lipid Desaturation

**Figure 6:**
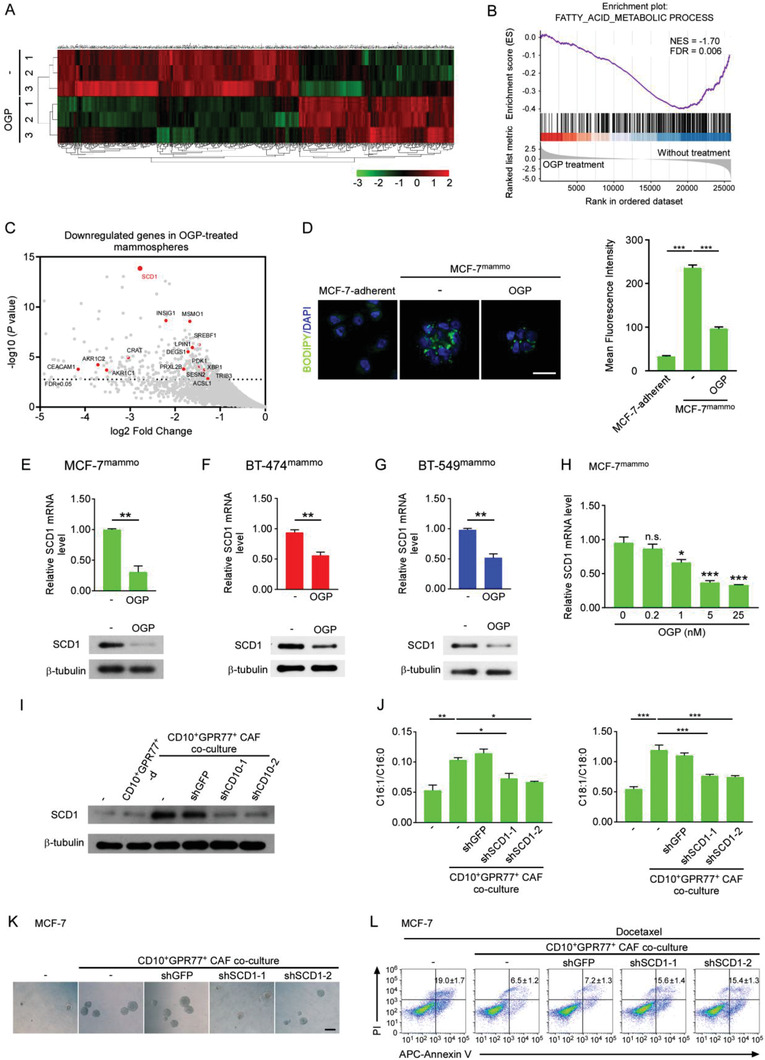
OGP suppresses CSCs by inhibiting lipid desaturation. A–C) MCF‐7^mammo^ cells were treated with or without 5 × 10^−9^ m OGP (*n* = 3). A) Heatmaps representing differentially expressed genes (fold change > 2, *P* < 0.05). B) Gene set enrichment analysis (GSEA)‐based analysis revealed a significant enrichment of the fatty acid metabolism‐related gene set in downregulated mRNAs after OGP treatment. C) Volcano plot showing downregulated genes in MCF‐7^mammo^ cells treated with OGP. Red dots represent top genes (log_2_ fold change > 2 and FDR < 0.05, compared to the control group) that were enriched in fatty acid metabolic process. The dashed line indicates an FDR of 0.05. D) Representative immunofluorescent images (left) and quantification (right) of BODIPY staining in MCF‐7 cells and MCF‐7^mammo^ cells treated with or without OGP. Scale bar, 20 µm. The quantification was made by calculating the fluorescence intensity of BODIPY in 6 randomly selected regions of interest for each sample. Mean ± SEM, *n* = 3. ***P < 0.001 by one‐way ANOVA. E‐G Levels of SCD1 in E) MCF‐7^mammo^, F) BT‐474^mamm^, and G) BT‐549^mammo^ cells treated with OGP were detected by qRT‐PCR (upper panel) and western blotting (lower panel) (mean ± SEM, *n* = 3). **P < 0.01 by Student's *t*‐test. H) The expression of SCD1 in MCF‐7^mammo^ cells treated with or without OGP at the indicated concentrations was determined by qRT‐PCR. Mean ± SEM, *n* = 3. N.s., not significant; *P < 0.05; ***P < 0.001 by one‐way ANOVA. I) Western blotting of SCD1 levels in MCF‐7 cells after coculture with CD10^+^GPR77^+^‐d CAFs or CD10^+^GPR77^+^ CAFs with CD10 knockdown (*n* = 3). J–L) MCF‐7 cells transduced with SCD1 shRNAs were cocultured with CD10^+^GPR77^+^ CAFs. J) Ratio of monounsaturated fatty acid to saturated fatty acid (C16:1/C16:0, C18:1/C18:0) in MCF‐7 cells determined by GC‐MS (mean ± SEM, *n* = 3). *P < 0.05; **P < 0.01; ***P < 0.001 by one‐way ANOVA. K) Representative images of mammosphere formation in MCF‐7 cells Scale bar, 100 µm. L) Representative flow cytometry plots indicate docetaxel‐induced apoptosis in MCF‐7 cells. Numerical values are presented as Annexin V^+^ percentage (mean ± SEM, *n* = 3).

To investigate the underlying mechanism of CSC suppression by OGP, the differentially expressed mRNAs between MCF‐7^mammo^ cells and those treated with OGP were identified (**Figure** **6**A). To gain insight into the potential biological processes and pathways mediated by OGP, the sequencing data were used for gene set enrichment analysis (GSEA). Among the top‐ranked pathways, a negative enrichment of genes involved in fatty acid metabolism was found in MCF‐7^mammo^ cells treated with OGP (False Discovery Rate (FDR) < 0.25; normalized enrichment score (NES) = −1.70) (Figure 6B,C; Figure S5A, Supporting Information). Consistently, 4,4‐difluoro‐1,3,5,7,8‐pentamethyl‐4‐bora‐3a,4a‐diaza‐s‐indacene (BODIPY) staining revealed a substantially reduced amount of lipid droplets in MCF‐7^mammo^ cells following OGP treatment (Figure 6D). Interestingly, the most significantly downregulated gene in fatty acid metabolic process, *SCD1*, encodes stearoyl‐coenzyme A desaturase‐1 (SCD1), which is recognized as a metabolic hallmark of CSCs.^[^ ^55^, ^56^, ^57^ ^]^ qRT‐PCR and western blotting further confirmed that OGP suppressed the expression of SCD1 in MCF‐7^mammo^ cells, BT‐474^mammo^, and BT‐549^mammo^ cells (Figure 6E–G). To explore whether OGP inhibits SCD1 expression via its bioactive domain, MCF‐7^mammo^ cells were treated with OGP and OGP (Y10A). A dose‐dependent decrease in SCD1 levels in tumor cells was observed after OGP treatment (Figure 6H). In contrast, OGP (Y10A) did not significantly affect SCD1 expression in tumor cells (Figure S5B, Supporting Information). Moreover, coculture with CD10^+^GPR77^+^ CAFs dramatically increased SCD1 expression in tumor cells, which could be reversed by CD10 knockdown (Figure 6I). These results indicate that OGP inhibits the expression of SCD1 in tumor cells via its bioactive domain, and that this effect is significantly abrogated by CD10 on CD10^+^GPR77^+^ CAFs.

SCD1 is a rate‐limiting enzyme that converts saturated fatty acids to monounsaturated fatty acids.^[^ ^58^ ^]^ To characterize lipid unsaturation in tumor cells cocultured with CD10^+^GPR77^+^ CAFs, gas chromatography–mass spectrometry (GC‐MS) analysis was performed. The ratio of monounsaturated fatty acids to saturated fatty acids (C16:1/C16:0 and C18:1/C18:0) significantly increased in MCF‐7 cells cocultured with CD10^+^GPR77^+^ CAFs. Importantly, transduction of shSCD1 abrogated the elevation of C16:1/C16:0 and C18:1/C18:0 fatty acid ratios induced by CD10^+^GPR77^+^ CAFs (Figure 6J). Furthermore, silencing SCD1 in tumor cells markedly reduced mammosphere generation (Figure 6K; Figure S5C–E, Supporting Information) and increased the proportion of apoptotic tumor cells exposed to chemotherapy (Figure 6L; Figure S5F, Supporting Information). Similar results were observed in BT‐474 and BT‐549 cells (Figure S5G–J, Supporting Information). To investigate the effect of OGP on other lipogenic factors, such as SREBP‐1 or FASN, the expression of SREBP‐1 and FASN was assessed in MCF‐7^mammo^ cells that were untreated or treated with OGP by qRT‐PCR and western blotting. In contrast to SCD1, no appreciable effect of OGP on SREBP‐1 or FASN was observed (Figure S5K, Supporting Information). Collectively, these data suggest that OGP reduces the expression of SCD1 and subsequently inhibits lipid desaturation, which are required for CSC maintenance.

### 2.5 OGP Suppresses SCD1 Expression by Inhibiting the NF‐*κ*B Pathway

**Figure 7:**
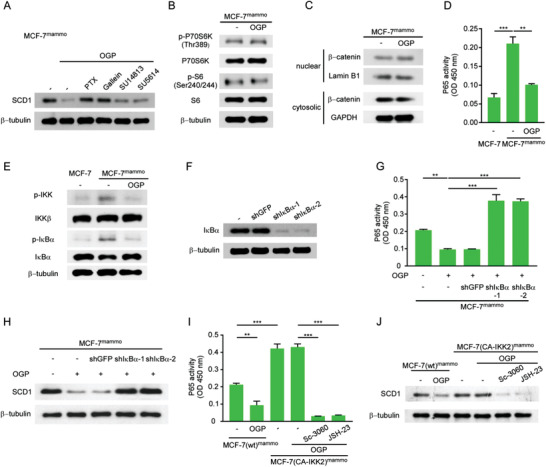
OGP suppresses SCD1 expression by inhibiting the NF‐*κ*B pathway. A) MCF‐7^mammo^ cells preincubated with or without indicated inhibitors were treated with OGP. The level of SCD1 was detected by western blotting (*n* = 3). B,C) MCF‐7^mammo^ cells were treated without or with OGP. B) Levels of total p70 S6 Kinase (P70S6K), S6 Ribosomal Protein (S6), and phosphorylated p70 S6 Kinase (p‐P70S6K), S6 Ribosomal Protein (p‐S6) were determined by western blotting (*n* = 3). C) Levels of intranuclear and cytosolic *β*‐catenin were determined by western blotting (*n* = 3). D,E) MCF‐7 cells and MCF‐7^mammo^ cells were treated without or with OGP. Nuclear proteins were analyzed for NF‐*κ*B DNA binding activity by D) ELISA and phosphorylated IKK, and I*κ*B*α* were detected by E) western blot analysis (*n* = 3). F) I*κ*B*α* levels in MCF‐7^mammo^ cells transduced with I*κ*B*α* shRNA (*n* = 3). G,H) MCF‐7^mammo^ cells with or without I*κ*B*α* knockdown were treated with or without OGP. G) Nuclear proteins were analyzed for NF‐*κ*B DNA binding activity by ELISA (*n* = 3). H) SCD1 levels were determined by western blotting (*n* = 3). I,J) MCF‐7^mammo^ cells transfected with (CA‐IKK2) or without (wt) plasmid expressing CA‐IKK2 were preincubated without or with indicated inhibitors before OGP treatment. I) Nuclear proteins were analyzed for NF‐*κ*B DNA binding activity by ELISA (*n* = 3). J) SCD1 levels were determined by western blotting (*n* = 3). D,G,I) Mean ± SEM, **P < 0.01; ***P < 0.001 by one‐way ANOVA.

We next investigated the mechanism by which OGP suppresses SCD1. Given that the receptor of OGP remains unidentified, MCF7^mammo^ cells were pretreated with G protein coupled receptor inhibitors, pertussis toxin (PTX) and gallein, or receptor tyrosine kinase inhibitors, SU14813 and SU5614, before OGP challenge. Immunoblots revealed that PTX and Gallein abrogated the downregulation of SCD1 in tumor cells treated with OGP, whereas SU14813 and SU5614 had little effect (**Figure** **7**A). To further dissect the biological pathways in CSCs modulated by OGP, MCF7^mammo^ cells were treated with OGP and the activation of mammalian target of rapamycin (mTOR), *β*‐catenin, and nuclear factor‐kappa B (NF‐*κ*B) pathways was evaluated. These pathways have been reported to mediate SCD1 expression.^[^ ^59^, ^60^, ^61^, ^62^, ^63^ ^]^ No significant changes in S6 and P70S6K phosphorylation by mTORC1 or *β*‐catenin nuclear translocation after OGP stimulation were observed (Figure 7B,C). In contrast, OGP treatment significantly suppressed NF‐*κ*B transcriptional activity (Figure 7D). Furthermore, phosphorylation of IKK*α*/*β* (Ser176/180) and I*κ*B*α* (Ser32) was greatly diminished in MCF7^mammo^ cells following OGP addition (Figure 7E). To investigate whether OGP inhibits SCD1 by suppressing NF‐*κ*B, I*κ*B*α* was silenced. The effects of OGP on NF‐*κ*B activation and SCD1 expression were abolished by I*κ*B*α* knockdown (Figure 7F–H). Given that IKK is the upstream regulatory factor of I*κ*B*α*, we investigated whether OGP inhibits NF‐*κ*B activation by suppressing the phosphorylation of IKK. Constitutively active IKK*β* (CA‐IKK2) was transfected.^[^ ^64^ ^]^ OGP inhibition of NF‐*κ*B transcriptional activity and SCD1 expression were abrogated (Figure 7I,J). This rescue effect was completely absent in MCF7^mammo^ cells treated with Sc‐3060 or JSH‐23, two inhibitors of NF‐*κ*B nuclear translocation. These data indicate that OGP suppresses the expression of SCD1 by inhibiting the phosphorylation of IKK and NF‐*κ*B activity.

### 2.6 Hydrolyzation of OGP by CD10 Provides a Potential Therapeutic Target for Cancer Treatment

**Figure 8:**
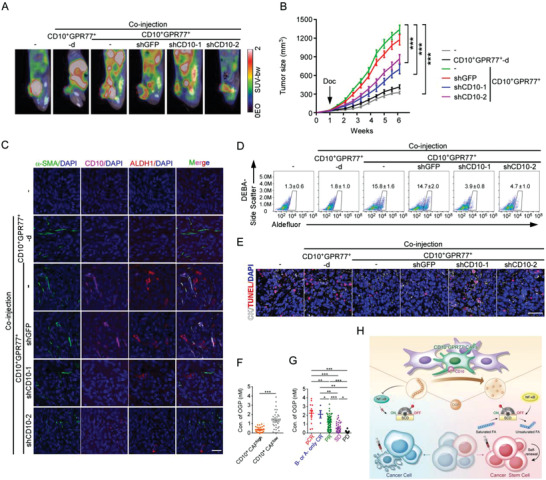
Hydrolyzation of OGP by CD10 provides a potential therapeutic target for cancer treatments. A–E) MCF‐7 cells alone (−) or with indicated CAFs were injected into fat‐pads of 6‐weeks‐old NOD/SCID mice. Docetaxel was administrated when tumors were palpable. A) Representative images of xenograft growth monitored by PET‐CT (*n* = 6 per group). The circles indicate xenografts. B) Tumor growth curves (*n* = 6 per group). Mean ± SEM, ***P < 0.001 by one‐way ANOVA at week 6. C) Representative images of *α*‐SMA, CD10, and ALDH1 immunofluorescence staining in the sections of xenografts (*n* = 6 per group). Scale bar, 50 µm. D) The proportions of ALDH1^+^ tumor cells isolated from harvested xenografts were determined by flow cytometry (mean ± SEM, *n* = 5 per group). E) Representative images of immunofluorescence staining for apoptotic tumor cells (TUNEL^+^CK^+^) in the sections of xenografts (*n* = 6 per group). F) OGP levels in tumor interstitial fluid of CD10^+^ CAF^high^ (*n* = 29) and CD10^+^ CAF^low/−^ (*n* = 35) human breast cancer samples were evaluated by ELISA (mean ± SEM). ***P < 0.001 by Mann‐Whitney U tests. G) Biopsies from patients with different responses to neoadjuvant chemotherapy were obtained before treatments. OGP levels in tumor interstitial fluid were evaluated by ELISA. pCR, pathological complete response, no invasive carcinoma or ductal carcinoma in situ in breast or axilla, *n* = 13; B‐ or A‐only pCR, pCR in breast or axilla only, *n* = 4; PR, partial remission, *n* = 62; SD, stable disease, *n* = 37; PD, progressive disease, *n* = 8. Mean ± SEM, *P < 0.05; **P < 0.01; ***P < 0.001 by Mann‐Whitney U tests. H) Schematics highlighting the primary findings of this study.

To evaluate the therapeutic potential of CD10‐OGP signals in vivo, we adopted a xenograft mouse model by coinjecting human CAFs and breast cancer cells into the mammary fat pads of immunocompromised mice, as previously described.^[^ ^25^ ^]^ Mice were administered chemotherapy after the tumors were palpable. Coinjection of MCF‐7 cells with CD10^+^GPR77^+^ CAFs instead of CD10^+^GPR77^+^‐depleted CAFs in mice markedly reduced chemotherapy efficacy, which was significantly improved by CD10 knockdown in CD10^+^GPR77^+^ CAFs (**Figure** **8**A,B; Figure S6A, Supporting Information). CD10^+^GPR77^+^ CAFs also significantly promoted ALDH1^+^ CSC enrichment in the coinjected cancer cells (Figure 8C,D; Figure S6B,C, Supporting Information). Consistently, coinjection of CD10^+^GPR77^+^ CAFs protected tumor cells from chemotherapy‐induced apoptosis in vivo (Figure 8E; Figure S6D, Supporting Information). Again, silencing CD10 markedly abrogated these effects (Figure 8C–E; Figure S6B–D, Supporting Information).

To investigate whether OGP cleavage may reduce its quantitation by the ELISA assay, the concentrations of full‐length OGP and those incubated with rhCD10 were determined. The concentration of OGP_1–14_ detected by ELISA was markedly decreased by rhCD10 treatment (Figure S6E, Supporting Information), indicating that OGP cleavage reduces its quantity. To evaluate the correlation between CD10 and OGP in clinical samples, the concentration of OGP in the interstitial fluid from tumors of breast cancer patients was determined by ELISA (Figure 8F,G). The level of OGP was significantly lower in clinical samples with high CD10^+^ CAF infiltration than in those with low infiltration (Figure 8F). In addition, the concentration of OGP in biopsies of responders was significantly higher than that in non‐responders before receiving neoadjuvant chemotherapy (Figure 8G), suggesting that OGP is associated with improved chemotherapy response in breast cancer patients. Taken together, these results suggest that blocking CD10 may be a potential therapeutic target for breast cancer.

## 3 Conclusion

Herein, we uncovered the functions and underlying mechanisms of CD10, a transmembrane hydrolase expressed by a subset of CAFs. CD10 is not only a cell surface marker but also a functional driver of tumor stemness and chemoresistance due to hydrolysis of an antitumor peptide. As a substrate of CD10 in tumors, OGP alters lipid metabolism in CSCs by inhibiting the expression of SCD1 desaturase (Figure 8H). The findings of the present study reveal a cell surface peptolytic signaling axis in the TME, which dictates the cell fate of CSCs by regulating their lipid metabolism, and provides the preclinical justification for the development of oncological therapies to block CD10.

CAFs play a crucial role in malignant development.^[^ ^2^, ^65^ ^]^ However, previous attempts to target total CAFs clinically failed to show any benefit.^[^ ^5^, ^7^, ^66^, ^67^ ^]^ The disappointing results of these clinical trials can be explained by the recent discovery that CAFs are composed of heterogeneous subpopulations with different or even opposite functions. For example, our previous study showed that a CD10^+^GPR77^+^ CAF subset enhances tumor progression via IL‐6 and IL‐8, which is initiated by the complement‐GPR77 signal.^[^ ^25^ ^]^ In contrast, another study reported an IKK*β*‐driven subset of CAFs expressing Col1a2, which suppresses intestinal tumor growth.^[^ ^68^ ^]^ Therefore, we and others have shown that the hurdle of CAF‐oriented therapy can be circumvented by selectively targeting specific tumor‐promoting CAF subsets. This emerging notion is further strengthened by our finding that CD10 in CAFs sustains tumor stemness of CSCs and targeting CD10 can enhance the efficacy of chemotherapy in vivo. Thus, this study improves the understanding of the functional heterogeneity and disparate roles of CAFs and provides experimental evidence that CD10 is a potential therapeutic target for breast cancer treatment. Future research is warranted to explore the clinical applications of CD10 blockade.

Here, we showed that a subset of fibroblasts maintains the properties of breast cancer stem cells (CSCs) via CD10. Interestingly, it has been reported that CD10 is also expressed on CSCs of other tumor types and is associated with chemoresistance,^[^ ^31^, ^32^ ^]^ although the underlying mechanisms remain unknown. Our data demonstrated that CD10 levels were much lower in breast cancer cells than in fibroblasts, suggesting that the CD10 expression profile in different cell types is heterogeneous among various tumor types. However, these previous observations can be fully explained by the mechanism uncovered here. Together, the findings of the present study and prior studies collectively highlight the importance of CD10 in the CSC niche. The previously unknown CD10 pathway revealed in the present study generates therapeutic opportunities to improve the efficacy of conventional treatment in a broad range of malignancies.

CAFs can drive tumor progression via cytokine secretion and immune checkpoint expression. For example, we^[^ ^25^ ^]^ and others^[^ ^69^, ^70^, ^71^ ^]^ have demonstrated that cytokines released by CAFs, including C‐X‐C motif chemokine 12, transforming growth factor‐*β*, IL‐6, and IL‐8, induce tumor metastasis and chemoresistance. In addition, CAFs induce immunosuppression by expressing immune checkpoints, including CD73, programmed death‐ligand 1 (PD‐L1), PD‐L2, and Fas ligand, which suppress CD4^+^ and CD8^+^ T cells in the TME.^[^ ^72^, ^73^, ^74^ ^]^ Here, we revealed that a transmembrane hydrolase expressed by a subset of CAFs sustains tumor stemness by degrading an antitumor peptide. Therefore, in conjunction with earlier reports, our results collectively indicate that CAFs can mediate tumor progression through multiple strategies.

By analyzing the interstitial fluid peptide profiles of breast cancer samples, we identified OGP as a key substrate of CD10 in breast cancer. CD10 cleaves small peptides of fewer than 43 amino acids. The consensus sequence for efficient hydrolysis by CD10 is ‐Phe‐Phe‐Gly‐|‐Phe‐Leu‐(Ala).^[^ ^75^ ^]^ Interestingly, Gly‐|‐Phe^[^ ^76^ ^]^ which has the highest rate of hydrolysis, matches the sequence of the bioactive domain in OGP (‐Tyr‐Gly Phe‐Gly Gly).

Metabolic reprogramming dictates cell fate.^[^ ^77^ ^]^ Emerging evidence has shown that lipid metabolism plays an important role in the generation and maintenance of CSCs.^[^ ^78^, ^79^ ^]^ Our study uncovered a previously unappreciated role of OGP in CSC suppression by reducing the generation of free monounsaturated fatty acids. OGP inhibits SCD1, a highly conserved Δ‐9 desaturase that converts saturated fatty acids to monounsaturated fatty acids.^[^ ^80^ ^]^ In agreement, previous studies have demonstrated that OGP hinders the expression of adipose‐specific genes. For example, OGP inhibits the expression of peroxisome proliferator‐activated receptor‐gamma 2 (PPAR*γ* 2) in adipocytes, which regulates adipogenic differentiation.^[^ ^81^ ^]^ Thus, our data reveal a nexus between the peptolytic signals in the TME and lipid metabolism in tumor cells to sustain tumor stemness.

Mechanistically, little is known about the intracellular signaling process initiated by OGP.^[^ ^37^, ^82^ ^]^ In the present study, OGP stimulated G protein coupled receptors on CSCs and subsequently downregulated the NF‐*κ*B pathway to suppress the expression of SCD1. Nevertheless, levels of other lipogenic factors, such as sterol regulatory element‐binding transcription factor 1 or fatty acid synthase in CSCs were not significantly altered upon OGP treatment, supporting the notion that OGP directly diminishes SCD1 expression by inhibiting NF‐*κ*B. Consistent with this, a previous study of ovarian CSCs showed that NF‐*κ*B p65 stimulates SCD1 expression by binding to its promoter region.^[^ ^55^ ^]^ However, the exact intracellular mechanism of NF‐*κ*B inhibition by OGP remains unclear, and future studies are warranted.

Interestingly, OGP is recognized as a growth factor for bone regeneration and repair.^[^ ^81^, ^83^ ^]^ Under normal circumstances, there is a balance between osteoblasts and adipocytes in the bone marrow that are maintained by MSCs. OGP promotes osteogenesis by promoting osteogenic maturation and inhibiting adipogenic differentiation of MSCs,^[^ ^81^ ^]^ although the underlying mechanisms remain largely unknown. These phenomena can be at least partially explained by our finding that OGP inhibits SCD1 and consequently impairs lipid desaturation, which is essential for adipogenic differentiation of MSCs.^[^ ^81^ ^]^ Thus, the role of SCD1 inhibition by OGP in manipulating the differentiation of other stem cells warrants further study.

In summary, the findings reveal a cell surface peptolytic signaling axis present in a tumor‐promoting CAF subset. The findings also raise the possibility that CAFs shape tumor cell fate by modulating their lipid metabolism. Clinically, the findings reveal a functional marker of CAFs as a targetable metabolic vulnerability of CSCs, which can be exploited to improve existing therapies.

## 4 Experimental Section

### Patients and Specimens

Fresh resected tumors and paired paraffin‐embedded samples were obtained from female patients with stage I–III primary breast cancer at the Sun Yat‐Sen Memorial Hospital, Sun Yat‐Sen University (Guangzhou, China) from 2017 to 2021. These cases included 73 samples for isolation of CAFs and 72 samples for extraction of tumor interstitial fluid (TIF). The latter 72 samples comprised 64 samples for detection of OGP concentration by ELISA and eight samples for peptidomics analysis.

Fresh biopsy samples for detection of OGP concentration in TIF by ELISA were obtained from 124 female patients with stage I–III primary breast cancer receiving neoadjuvant chemotherapy. For Her2‐negative patients, the neoadjuvant chemotherapy regimens comprised doxorubicin (60 mg m^−2^) plus cyclophosphamide (600 mg m^−2^) every 2 weeks for four cycles, followed by paclitaxel (175 mg m^−2^) every 2 weeks for four cycles, paclitaxel (80 mg m^−2^) every week for 12 weeks, or cyclophosphamide (600 mg m^−2^) and docetaxel (75 mg m^−2^) every 3 weeks for four cycles. For Her2‐positive patients, four cycles of doxorubicin at a dose of 60 mg m^−2^ and cyclophosphamide at a dose of 600 mg m^−2^ were administrated every 21 days, with a subsequent weekly paclitaxel (80 mg m^−2^) treatment for 3 months. Patients concomitantly received Herceptin at an initial dose of 4 mg kg^−1^ followed by subsequent doses of 2 mg kg^−1^ for a weekly schedule or 6 mg kg^−1^ for a three‐weekly schedule for one year.

Response Evaluation Criteria in Solid Tumors (RECIST) guidelines were applied to assess the patients’ responses to neoadjuvant treatment. Pathological complete response (pCR) refers to the absence of any residual invasive or in situ carcinoma in the breast or lymph node. Breast/node‐only pCR refers to pCR limited to either breast or axilla. Partial response refers to a ≥30% reduction in tumor size. Progressive disease (PD) refers to a ≥20% enlargement of the tumor. Stable disease (SD) refers to a range from <20% growth to <30% reduction in tumor size.

### Isolation and Culture of Human CAFs

Primary CAFs were isolated from fresh breast cancer samples as previously described.^[^ ^25^ ^]^ Briefly, the tumor tissues were minced into pieces 1–2 mm in diameter and digested by collagenase I (1.5 mg mL^−1^, Worthington), collagenase III (1.5 mg mL^−1^, Worthington), and hyaluronidase (1.5 mg mL^−1^, Sigma‐Aldrich) in DMEM containing 10% fetal bovine serum (FBS) at 37 °C with agitation for 3 h. The solution was incubated at 26 °C for 5 min. The stromal cell‐enriched supernatant was transferred into a new tube and centrifuged at 250 × *g* for 5 min. After the supernatant was removed, the pellet was resuspended and cultured in DMEM supplemented with 20% FBS for fibroblast growth.^[^ ^70^ ^]^

CD10^+^GPR77^+^ CAFs and CD10^+^GPR77^+^‐depleted CAFs were sorted by fluorescence‐activated cell sorting using a flow cytometer (BD Influx) as previously described.^[^ ^25^ ^]^ To exclude dead cells, the cells were stained using a live/dead viability dye (FVD‐eFluor780, Cat# 65‐0865‐18, eBioscience) in PBS for 30 min at 4 °C. After washing with PBS, CAFs were incubated with antibodies against anti‐CD45‐FITC (Cat# 304 006, BioLegend), anti‐CD31‐PE/Cy7 (Cat# 303 118, BioLegend), anti‐EpCAM‐PerCP/Cy5.5 (Cat# 324 214, BioLegend), anti‐CD10‐APC (Cat# 17‐0106‐42, eBioscience), and anti‐GPR77‐PE (Cat# 342 404, BioLegend) in PBS containing 1% FBS for 30 min at 4 °C. After washing with PBS three times, the cells were resuspended in PBS and examined by flow cytometry. Purity was assessed by flow cytometry. CAFs from passages two to nine were used for the subsequent experiments.

### Flow Cytometry

For cell surface marker analysis, cells were resuspended in PBS containing 1% FBS and incubated with CD24‐FITC (Cat# 11‐0247‐42, eBioscience) and CD44‐APC (Cat# 17‐0441‐81, eBioscience) for 30 min at 4 °C. Aldehyde dehydrogenase 1 (ALDH1) activity of cells was detected using the ALDEFLUOR kit (Cat# 0 1700, Stem Cell Technologies) according to the manufacturer's instructions. The samples were analyzed using a CytoFLEX flow cytometer (Beckman‐Coulter). Data were analyzed by FlowJo software.

### Immunofluorescence

Primary tumor tissues were embedded in paraffin and sectioned into 4 µm‐thick sections. Antigen retrieval was performed using sodium citrate buffer (pH 6.0). Sections were blocked in PBS containing 2% BSA at 26 °C for 1 h. The specimens were then treated with antibodies against *α*‐SMA (Cat# MAB1420, R&D, 1:100; Cat# ab21027, Abcam, 1:100), CD10 (Cat# ab73409, Abcam, 1:50), GPR77 (Cat# 342 402, BioLegend, 1:50), or ALDH1 (Cat# AF5869, R&D, 1:100) overnight at 4 °C. After washing with PBS, specimens were incubated with secondary antibodies conjugated to fluorescent dyes (Cat# A32814, Cat# A21202, Cat# A31573, Cat# A31570, Cat# A32816, Thermo Fisher Scientific) for 1 h at 26 °C. 4′,6‐Diamidino‐2‐phenylindole (DAPI; Cat# D3571, Thermo Fisher Scientific) was used to stain the nuclei of cells. Immunofluorescent images were acquired using a LSM780 confocal microscope (Carl Zeiss). The percentage of CD10^+^ CAFs was assessed staining of CD10 and alpha‐smooth muscle actin (*α*‐SMA) in tumor sections and automated analysis with Imaris 9.0 software.^[^ ^84^ ^]^ Cell number was quantified by calculating the DAPI^+^ spots. The number of *α*‐SMA^+^CD10^+^ spots was divided by that of *α*‐SMA^+^ spots in the sample to obtain the proportion of CD10^+^ CAFs.

### Peptide Synthesis

Peptides including OGP_1–14_, OGP_1–9_, OGP_10–14_, OGP (L2A), OGP (Y10A), and biotinylated OGP were synthesized by Focus Biology (Nanchang, China). Peptides prepared according to a standard solid‐phase synthesis strategy^[^ ^85^ ^]^ were purified by reverse‐phase high‐pressure liquid chromatography (RP‐HPLC) (Shimadzu) on a C18 column (150 mm × 20 mm, 10 µm particle size, Higgins Analytical) and lyophilized. Identities of the peptides were confirmed by matrix‐assisted laser desorption/ionization‐time of flight (MALDI‐TOF) mass spectrometry (Waters) using *α*‐cyano‐4‐hydroxycinnamic acid as the matrix. The peptides were resolved in sterile PBS before use.

### Coculture Experiments

MCF‐7, BT‐549, BT‐474, and SK‐BR3 breast cancer cells obtained from the American Type Culture Collection were cultured according to standard protocols. For coculture experiments, 1 × 10^5^ breast cancer cells and 1 × 10^5^ fibroblasts were seeded into the lower and upper chambers of a six‐well Transwell apparatus with pore size of 0.4 µm (Corning Incorporated).^[^ ^25^ ^]^ Cells were passaged once they reached nearly 90% confluence. In some experiments, 500 × 10^−9^ m OGP (L2A) or OGP (Y10A) was added to the coculture system.^[^ ^49^, ^51^ ^]^ After 2 weeks of coculture, the cells were subjected to further analysis. In some experiments, tumor cells were cocultured with fibroblasts in HuMEC basal serum‐free medium (Cat# 12 753 018, Thermo Fisher Scientific), which is free of both serum and BSA.^[^ ^86^, ^87^ ^]^

### Lentiviral VectorMediated Silencing or Overexpression

Lentivirus vector LV3‐pGLV‐H1‐GFP/Puro was used for knockdown experiments. The shCD10‐1 and shCD10‐2 sequences were 5′‐GCUAUUGCACAACUGAAUUTT‐3′ and 5′‐AAUUCAGUUGUGCAAUAGCTT‐3′, respectively. The shSCD1‐1 and shSCD1‐2 sequences were 5′‐CTACGGCTCTTTCTGATCA‐3′ and 5′‐GGAGAATATCCTGGTTTCA‐3′, respectively. The shI*κ*B*α*‐1 and shI*κ*B*α*‐2 sequences were 5′‐CCAUGAAAGACGAGGAGUA‐3′ and 5′‐GCGACGGGCUGAAGAAGGA‐3′ respectively. For CD10 overexpression, the cloned CD10 cDNA was inserted into the LV5‐pGLV‐EF1a‐GFP/Puro lentiviral plasmid vector. Lentiviral vectors were designed and constructed by GenePharma Inc. (Suzhou, China). For transduction of primary fibroblasts, 1 × 10^6^ cells were transduced with lentiviral particles (multiplicity of infection, MOI, of 100) overnight with 8 µg mL^−1^ polybrene (Sigma‐Aldrich). For transduction of tumor cells, 1 × 10^6^ cells were transduced with lentiviral particles (MOI of 20) overnight with 8 µg mL^−1^ polybrene.

### Sphere Formation Assay

Mammosphere medium was prepared by mixing DMEM/F12 (Cat# C11330500BT, GIBCO), 0.4% BSA (Cat# A1933, Sigma‐Aldrich), 2% B27 (Cat# 17 504 044, Thermo Fisher Scientific), 20 ng mL^−1^ epidermal growth factor (EGF; Cat# 100‐47, PeproTech), and 4 mg mL^−1^ insulin (Cat# I9278, Sigma‐Aldrich). In some experiments, tumor cells were cultured in a BSA‐free mammary epithelial growth medium (BioWhittaker) supplemented with 5 µg mL^−1^ insulin, 0.5 µg mL^−1^ hydrocortisone, 2% B27 (Cat# 17 504 044, Thermo Fisher Scientific), 20 ng mL^−1^ EGF and basic fibroblast growth factor (bFGF; BD Biosciences), and 4 µg mL^−1^ heparin (Sigma‐Aldrich) as previously reported to exclude the effects of BSA.^[^ ^53^, ^88^ ^]^ Cancer cells in mammosphere medium (10^3^ cells per mL) were seeded in ultra‐low adhesion plates and cultured at 37 °C for 10 days. Mammospheres exceeding 75 µm in diameter were counted.

In some experiments, mammospheres were serially cultured for 5 weeks to enrich CSCs, as previously described.^[^ ^43^, ^89^ ^]^ Afterwards, cells were treated with or 5 × 10^−9^ m OGP_1–14_, OGP_10–14_, OGP_1–9_, OGP(L2A), or OGP(Y10A) for 10 days.^[^ ^35^, ^37^, ^40^, ^90^ ^]^ In some experiments, cells were treated with 5 × 10^−9^ m OGP_1–14_ in the presence or absence of 5 µg mL^−1^ recombinant human CD10 (rhCD10; Cat# ab157051, Abcam) for 10 days.^[^ ^91^, ^92^, ^93^ ^]^ Mammospheres exceeding 75 µm in diameter were counted and a chemotherapy‐induced apoptosis assay was performed.

In some experiments, MCF‐7^mammo^ cells were pretreated with 1 µg mL^−1^ pertussis toxin (Cat#P7208, Sigma‐Aldrich), 20 × 10^−6^ m Gallein (Cat# 371 708, Sigma‐Aldrich), 1 × 10^−6^ m SU14813 (Cat#S0504, Selleck), and 100 × 10^−9^ m SU5614 (Cat# S0278, Selleck) for 24 h, or with 10 × 10^−6^ m Sc‐3060 (Cat# Sc‐3060, Santa Cruz Biotechnology) or 6 × 10^−6^ m JSH‐23 (Cat# S7351, Selleck) for 1 h.

### Symmetrical Division Assessment

PKH26 was a fluorescent dye that binds to cell membranes and segregates in daughter cells after each cell division.^[^ ^53^ ^]^ Numb is one of the most important cell fate determinants and tends to segregate into differentiated cells during division.^[^ ^94^, ^95^ ^]^ When cells divide symmetrically, the fluorescent intensity of PKH26 dye in the two daughter cells was equal, and Numb symmetrically segregates into daughter cells. Otherwise, unequal levels of PHK26 dye and asymmetric distribution of Numb in daughter cells can be observed.

For PKH26 staining, tumor cells were dissociated and labeled with PKH26 (MINI26, Sigma‐Aldrich) as previously described.^[^ ^96^, ^97^ ^]^ Briefly, PKH26 staining solution was prepared by diluting PKH26 dye with Diluent C (1:250). Then, 5 × 10^6^ cells in 0.5 mL Diluent C were mixed with the staining solution (0.5 mL) and incubated at 26 °C for 1 min. Next, 1 mL of FBS was added to stop the staining process. PKH26‐labeled cells were washed with PBS and resuspended in mammosphere medium (1000 cells mL^−1^). The resuspended cells were seeded in six‐well ultra‐low‐attachment plates. After culturing for 3 days, the cells were collected and imaged using the LSM780 confocal microscope (Carl Zeiss).

For Numb staining, tumor cells were fixed with 4% paraformaldehyde for 15 min at 26 °C. Fixed cells were washed, permeabilized, and blocked as previously described.^[^ ^25^, ^98^ ^]^ Afterwards, the specimens were treated with anti‐Numb antibody (Cat# ab14140, Abcam, 1:50) for 10 h at 4 °C and incubated with an immunofluorescent secondary antibody (Cat# A21206, Thermo Fisher Scientific) for 1 h at 26 °C. The cells were then stained with DAPI (Cat# D3571, Thermo Fisher Scientific) and immunofluorescent images were acquired.

### Apoptosis Analysis

Tumor cells were treated with 20 ng mL^−1^ docetaxel for 12 h and digested with 0.25% trypsin. Apoptosis was detected using the Annexin V Apoptosis Detection Kit with propidium iodide (Cat# 640 932, Biolegend) following the manufacturer's instructions. Briefly, cells were washed with PBS, and then incubated with 100 µL of Annexin V binding buffer containing 5 µL of APC‐conjugated Annexin V and 10 µL of propidium iodide solution for 15 min at 26 °C. Next, 400 µL of Annexin V Binding Buffer was added to each sample, and the cells were immediately analyzed by flow cytometry.

### Western Blot

Cells were collected and washed with PBS three times. Ripa lysis buffer (Millipore) containing a proteinase inhibitor cocktail (Cat# 78 446, Thermo Fisher Scientific) was added to the cells, and the suspensions were homogenized on ice using an electric homogenizer. Cell lysates were examined by 12% SDS‐PAGE. The nitrocellulose membranes were blocked and incubated with anti‐caspase‐3 (Cat# 9662, Cell Signaling Technology, 1:1000), anti‐cleaved caspase‐3 (Cat# 9664, Cell Signaling Technology, 1:1000), anti‐total poly (ADP‐ribose) polymerase (PARP) and anti‐cleaved PARP (Cat# 9542, Cell Signaling Technology, 1:1000), CD10 (Cat# ab73409, Abcam, 1:1000), GPR77 (Cat# 342 402, Biolegend), SCD1 (Cat# 2794, Cell Signaling Technology, 1:1000), S6 Ribosomal Protein (Cat# 2317, Cell Signaling Technology, 1:1000), phospho‐S6 Ribosomal Protein (Ser240/244) (Cat# 5364, Cell Signaling Technology, 1:1000), p70 S6 Kinase (Cat# 2708, Cell Signaling Technology, 1:1000), phospho‐p70 S6 Kinase (Thr389) (Cat# 9234, Cell Signaling Technology, 1:1000), I*κ*B*α* (Cat# 4814, Cell Signaling Technology, 1:1000), p‐I*κ*B*α* (Ser32) (Cat# 2859, Cell Signaling Technology, 1:1000), IKK*β* (Cat# 8943, Cell Signaling Technology, 1:1000), p‐IKK*α*/*β* (Ser176/180) (Cat# 2697, Cell Signaling Technology, 1:1000), SREBP1 (Cat# sc‐365513, Santa Cruz, 1:1000), FASN (Cat# 3180, Cell Signaling Technology, 1:1000), *β*‐catenin (Cat# sc‐7963, Santa Cruz, 1:1000), Lamin B1 (Cat# 12987‐1‐AP, Proteintech, 1:5000), *β*‐tubulin (Cat# 10068‐1‐AP, Proteintech, 1:2000), or glyceraldehyde 3‐phosphate dehydrogenase (GAPDH; Cat# HRP‐60004, Proteintech, 1:10 000). Membranes were incubated with peroxidase‐conjugated anti‐rabbit antibody (Cat# 7074, Cell Signaling Technology; 1:3000) or anti‐mouse antibody (Cat# 7076, Cell Signaling Technology; 1:3000) for 1 h at 26 °C. After washing with Tris buffered saline‐Tween five times, immunoreactive bands were visualized using SuperSignal West Femto (Cat#34 095, Thermo Fisher Scientific) in iBright imaging systems or X‐ray film.

### Peptidomics—Tumor interstitial fluid extraction

TIF was extracted as previously described.^[^ ^99^, ^100^, ^101^ ^]^ Briefly, fresh tumor tissue was rinsed with saline and blotted gently with tissue paper to remove excess saline. The tissue was placed onto a 20 µm nylon mesh membrane on a 50 mL conical centrifuge tube. The tube was centrifuged at 400 × *g*, a speed that was previously demonstrated not to liberate intracellular contents,^[^ ^99^, ^100^ ^]^ for 10 min at 4 °C to obtain TIF. TIF was snap‐frozen in liquid nitrogen and stored at −80 °C until further use.

### Peptide Isolation

Peptide separation was performed as previously described^[^ ^102^, ^103^ ^]^ with slight modifications. Briefly, a protease inhibitor cocktail was added to TIF (1:100) immediately before use. Acetonitrile (ACN) was then added (20% final v/v) to TIF, and the TIF was centrifuged at 20 000 × *g* at 4 °C for 10 min. Supernatant containing endogenous peptides was dispensed into an Ultra 1.5 mL 10 kDa molecular weight cut off (MWCO) centrifugal filter (Amnion). The MWCO filter was centrifuged at 14 000 × *g* for 30 min at 4 °C. The flow‐through was concentrated using a SpeedVac concentrator (Thermo Electron Corporation) and then resuspended in 20 µL of 0.1% formic acid (FA). The resulting sample was desalted using C18 ZipTips according to the manufacturer's instructions.^[^ ^104^ ^]^ Briefly, the ZipTips were conditioned with 100% ACN and equilibrated three times with 0.1% FA. Samples were dispensed onto the ZipTips slowly for at least 15 cycles and washed with 0.1% FA. Afterwards, peptides bound to the ZipTips were eluted with 20 µL of 50% ACN in 0.1% FA. The solution was dried and resuspended in 10 µL of 0.1% FA. The concentration of peptides was quantified using the Pierce™ quantitative colorimetric peptide assay (Thermo Fisher Scientific).

### LC‐MS/MS

Peptidomic profiling was performed using an Easy nLC 1200 system with an Orbitrap Fusion mass spectrometer, as previously described.^[^ ^105^ ^]^ Separation of the peptides was performed with a 75 µm × 150 mm capillary column packed with stationary phase (1.9 µm particle size, C18 AQ) with a gradient of 5–35% ACN containing 0.1% FA over 90 min at 300 nL min^−1^.^[^ ^106^ ^]^ An MS1 scan was acquired from 350 to 1800 m/z (120000 resolution, 4e5 AGC, 50 ms injection time) followed by MS/MS data‐dependent acquisition with EThcD and detection in the Orbitrap (50000 resolution, 5e4 AGC, 86 ms injection time, calibrated charge‐dependent ETD reaction times 60 ms, 30 NCE for HCD supplemental activation and 2.0 m/z quadrupole isolation width).

### Data Processing

Proteome Discoverer (v2.3.0) was applied for peptidomic data analysis using Andromeda against an in‐house constructed database based on the endogenous regulatory oligopeptide knowledgebase (EROP) of *Homo sapiens* (release 03_2020) containing 790 peptides. For the analysis, the precursor MS and EThcD MS/MS tolerance were set to 10 ppm and 0.02 Da, respectively. Peptide searching was performed as previously described.^[^ ^107^ ^]^ The FDR for the peptides was set to 0.01. Label‐free quantification (LFQ) in Proteome Discoverer was used to calculate the peptidomic data.

Peptidomics data have been uploaded to the ProteomeXchange Consortium.^[^ ^108^ ^]^ The identifiers were PXD021590 an https://doi.org/10.6019/PXD021590.

### Preparation of Hydrolysis Products and HPLC Analysis

OGP was synthesized as described above and resolved in water (1 × 10^−3^ m). OGP (4 µL) was incubated with or without 0.5 µL of rhCD10 (Cat# ab157051, Abcam) in a final volume of 200 µL of 50 × 10^−3^ m Tris‐HCl buffer (pH 7.5) for 1 h at 37 °C. To inhibit the enzymatic activity of rhCD10, rhCD10 was pretreated with 1 × 10^−6^ m thiorphan (Cat# ab141169, Abcam) for 30 min before incubation with OGP. The hydrolysis reaction was stopped by heating at 100 °C for 5 min. The peptide products were identified by reverse phase‐HPLC on a Phenomenex‐Pack Gemini‐NX 3 *μ* C18 110 Å (2.00 mm × 150 mm, 3 µm, Cat# 00F‐4453‐B0) column with an ultraviolet detector set at 214 nm.^[^ ^76^, ^109^, ^110^ ^]^ The peptides were resolved using a mobile phase consisting of 5% solvent A and 95% solvent B for 0.5 h at a velocity of 200 µL min^−1^ (solvent A = H_2_O, adjusted pH to 10.0 using NH_3_·H_2_O; solvent B = 80% ACN, adjusted pH to 10.0, using NH_3_·H_2_O).

### Determination of the Cleavage Sites of CD10 on OGP by HPLC‐MS/MS

OGP (4 µL) was incubated with or without 0.5 µL of rhCD10 (Cat# ab157051, Abcam) in a final volume of 200 µL of 50 × 10^−3^ m Tris‐HCl buffer (pH 7.5) for 1 h at 37 °C. To detect the cleavage sites of CD10 on OGP, enzymatic hydrolysis products of OGP after incubation with rhCD10 were used for HPLC‐MS/MS analysis.^[^ ^111^ ^]^ Briefly, the peptides were first loaded onto peptide trap columns (300 µm × 5 mm, 5 µm, Cat# 160 454, Thermo Fisher Scientific) and then separated on analytical columns (75 µm × 150 mm, 3 µm, Cat# 160 321, Thermo Fisher Scientific). Peptides bound to the columns were eluted with ACN in water (4–72% (v/v)) containing 0.1% FA at a flow rate of 300 nL min^−1^ for 40 min. The MS analysis was performed online with an Orbitrap spectrometer (Thermo Scientific Q Exactive; Thermo Fisher Scientific). Full‐mass and MS/MS scans were acquired as previously described.^[^ ^112^ ^]^

Peak list files were analyzed against an in‐house constructed database based on the sequence of OGP using Mascot software. Peptides with at least four amino acids were included in the analysis. The FDR for the peptides was set to 0.05.

### Sequence Alignment

The amino acid sequences of histone H4 were obtained from the UniProt database (http://www.uniprot.org/). The alignment of histone H4 and OGP sequences was conducted using *Clustal Omega*.^[^ ^47^, ^113^ ^]^

### Competitive Binding Assay

Tumor cell mounting slides were incubated with 100 × 10^−9^ m biotinylated OGP with or without 10 × 10^−6^ m OGP (L2A) or OGP (Y10A) in 0.01 mol L^−1^ Tris (pH 7.5) containing 1% bovine serum albumin for 1 h at 37 °C. After washing, cells were incubated with 10 µg mL^−1^ neutravidin‐Texas red (Cat# A2665, Thermo Fisher Scientific) in 0.01 mol L^−1^ Tris (pH 7.5) containing 0.5% BSA for 30 min at 26 °C.^[^ ^114^, ^115^ ^]^ For immunofluorescence analysis, the cells were washed with PBS and fixed with PBS‐buffered formalin for 10 min. Fixed cells were counterstained with DAPI and imaged using the LSM780 confocal microscope (Carl Zeiss). For flow cytometry analysis, the tumor cells were digested and washed with PBS. OGP‐mediated neutravidin‐Texas red uptake in cells was analyzed by flow cytometry.

### BODIPY Staining of Lipid Droplets

Cells were fixed with 3% formaldehyde for 15 min and then washed with PBS. Cells were then stained with BODIPY 493/503 (Cat# D3922, Invitrogen, 1 µg mL^−1^ in PBS) for 15 min at 26 °C. DAPI was used to label the nuclei, and images were obtained using the LSM780 confocal microscope (Carl Zeiss). BODIPY staining was quantified by calculating its mean fluorescence intensity in six randomly selected regions of interest (ROI) for each specimen using ImageJ.

### Human Serum Isolation

Peripheral blood samples from healthy donors were collected from the Guangzhou Blood Center. Human serum was isolated as described previously.^[^ ^84^ ^]^ Briefly, the coagulated peripheral blood was centrifuged at 2000 × *g* for 10 min at 4 °C. Afterwards, the upper layer serum was gently pipetted and removed into EP tubes. Serum was stored at −80 °C before the concentration of OGP was determined by ELISA. All specimens were tested for human immunodeficiency virus, syphilis, hepatitis B virus, and hepatitis C virus.

### ELISA

Human serum and TIF were obtained as described previously. Bovine serum was purchased from Gibco (Cat# 16000‐044). Serum and TIF were diluted with double distilled water (1:200) and incubated at 100 °C for 10 min to release OGP from the OGP and OGP binding protein (OGPBP) complex.^[^ ^46^ ^]^ The samples were cooled to 26 °C, centrifuged at 10 000 × *g* for 10 min at 4 °C, and supernatants were collected.

For the detection of IL‐6 and IL‐8, fibroblasts were maintained in DMEM with 10% FBS until the cells reached 80% confluency. The cells were washed with PBS and cultured in serum‐free DMEM. Supernatants were collected 24 h later, centrifuged to remove debris, and used for the subsequent ELISA assay. Levels of OGP (Cat# MBS2505219, MyBioSource), IL‐6 (Cat# 88‐7066‐86, eBioscience), and IL‐8 (Cat# 88‐8086‐86, eBioscience) were measured using ELISA kits according to the manufacturer's instructions. Optical density (OD) was measured spectrophotometrically at 450 nm. The concentrations of OGP, IL‐6, and IL‐8 in the samples were determined by comparing the OD of the samples to the standard curve.

### RNA Sequencing (RNA‐Seq) Library Preparation

Total RNA was extracted from MCF‐7^mammo^ cells treated with 5 × 10^−9^ m OGP for 10 days using TRIzol reagent (Invitrogen) according to the manufacturer's instructions. The RNA quantity and purity of each sample were determined using a Bioanalyzer 2100 and RNA 6000 Nano LabChip kit (Agilent). The RNA integrity number of each sample was over 7.0. mRNAs were purified using poly T oligo‐attached magnetic beads (Invitrogen) and then fragmented. The cleaved RNA fragments were reverse transcribed to create a cDNA library according to the manufacturer's protocol. The average insert size for the final cDNA library was 300 bp (±50 bp). Then, paired‐end sequencing was performed on an Illumina NovaSeq 6000 (LC Bio) following the manufacturer's protocol. The constructed RNA‐seq libraries were sequenced by the Lianchuan Corporation.

### Data Processing

Illumina sequence data were preprocessed as previously described to discard low‐quality reads.^[^ ^116^ ^]^ The sequenced reads were aligned to the human GRCh38 reference genome using the HISAT2 package (v.2.0.4). Fragments per kilobase of transcript per million mapped reads (FPKM) were calculated using StringTie (v.1.3.0) software. The differentially expressed mRNAs and genes were selected with fold change > 2 and were statistically significant (*P* < 0.05).^[^ ^117^ ^]^ GSEA was performed using the OmicStudio tools (https://www.omicstudio.cn/tool). The NES and FDR were calculated for comparison. Raw RNA sequencing data have been uploaded to the GEO database (accession number: GSE156470).

### qRT‐PCR

SYBR Premix Ex Taq II (Cat# RR820A, TaKaRa Bio) was used for routine real‐time PCR according to the manufacturer's instructions. The primer sequences were as follows: CD10‐forward: 5′‐TGGATCTTGTAAGCAGCCTCA‐3′; reverse, 5′‐GCACAACGTCTCCAAGTTGC‐3′; SCD1‐forward: 5′‐GTACCGCTGGCACATCAACTT‐3′, reverse: 5′‐TTGGAGACTTTCTTCCGGTCAT‐3′; SREBP1‐forward: 5′‐ ACTTCTGGAGGCATCGCAAGCA‐3′, reverse: 5′‐AGGTTCCAGAGGAGGCTACAAG‐3′; FASN‐forward: 5′‐TTCTACGGCTCCACGCTCTTCC‐3′, reverse: 5′‐ GAAGAGTCTTCGTCAGCCAGGA‐3′; and GAPDH‐forward: 5′‐GGAGCGAGATCCCTCCAAAAT‐3′, reverse: 5′‐GGCTGTTGTCATACTTCTCATGG‐3′. Gene expression was quantified using a LightCycler 480 instrument (Roche).

### MS Analysis of Fatty Acids

Fatty acids were extracted, derivatized, and analyzed by GC‐MS as previously described.^[^ ^118^, ^119^, ^120^ ^]^ Lipids were extracted from 2 × 10^6^ cells using methanol/water/chloroform. Briefly, the medium was removed and the cells were rinsed with saline. Cell metabolism was quenched by sequentially adding 500 µL of methanol at −80 °C and 200 µL of ice‐cold water. The cells were scraped into the solution and transferred to Eppendorf tubes. D31‐Palmitate solution (1 µg in 500 µL of −20 °C chloroform) was added to the samples as an internal standard. Tubes were vortexed for 15 min and centrifuged at 14 000 × *g* for 10 min at 4 °C. The bottom organic layer (lipids) was transferred to new tubes and dried under the flow of nitrogen. The dried lipids were dissolved in 500 µL of 2% (v/v) methanolic sulfuric acid and incubated at 50 °C for 2 h to generate fatty acid methyl esters (FAME). FAMEs were extracted in 1 mL hexane with 0.1 mL saturated sodium chloride, followed by evaporation under the flow of nitrogen.

The dried FAMEs were dissolved in 100 µL of hexane. In the splitless mode, 1 µL of the sample was injected into the Trace 1300 GC‐MS equipment (Thermo Fisher Scientific) with a 30 m DB‐35 ms UI column (Agilent Technologies) connected to a ISQ QD MS device (Thermo Fisher Scientific).^[^ ^118^ ^]^ Helium was used as the carrier gas at a flow rate of 1.5 mL min^−1^. The initial GC oven temperature was held at 100 °C for 3 min, then increased to 205 °C at a rate of 3.5 °C min^−1^ and ramped up to 230 °C at a rate of 0.5 °C min^−1^, followed by an increase to 300 °C at a rate of 25 °C min^−1^. The total run time was approximately 86 min. The injector and GC‐MS transfer line were maintained at 250 and 300 °C, respectively. Total ion chromatograms were acquired in the m/z range of 100–650. The peak areas of FAMEs identified with standard compounds were quantified with Xcalibur and TraceFinder 3.3 SP1 GQ software programs and normalized using D31‐Palmitate as an internal standard. The levels of C16:1, C16:0, C18:1, and C18:0 were measured by FAMEs derived from palmitoleate, palmitate, oleate, and stearate, respectively.^[^ ^118^ ^]^

### Plasmid Transfection

For NF‐*κ*B activation, MCF‐7^mammo^ cells were transfected with a plasmid for IKK*β* overexpression (CA‐IKK2, constitutively active IKK*β*), which has been used in the previous study.^[^ ^121^ ^]^ CA‐IKK2 was generated by substitution of Ser177 and Ser181 with glutamate as previously described by others as well.^[^ ^64^, ^121^ ^]^ Transfection was performed using Lipofectamine 3000 following the manufacturer's instructions.

### Coinjection Animal Experiments

Six‐week‐old NOD/SCID mice were injected under their fat pads with MCF‐7 cells (1 × 10^6^) or MCF‐7 cells (1 × 10^6^) mixed with CAFs (3 × 10^6^) as previously described.^[^ ^25^, ^70^ ^]^ To support the estrogen‐dependent MCF‐7 tumor growth, 17*β*‐estradiol 60‐day release pellets (Innovative Research of America) (1.7 mg per mouse) were subcutaneously implanted into mice 3 days prior to tumor injection. Mice were treated with docetaxel at a dose of 10 mg kg^−1^ by intraperitoneal injection weekly when the tumor reached a diameter of 3 mm.^[^ ^25^ ^]^ Tumor size was measured every 4 days with calipers, and the volume was calculated as 0.5 × length × width^2^. After 5 weeks of docetaxel treatment, positron emission tomography/computed tomography (PET/CT) scanning was performed. Next, the xenografts were harvested and subjected to further analysis.

To qualify ALDH1^+^ tumor cells in xenografts, the xenografts were dissociated by collagenase type I (1.5 mg mL^−1^) and collagenase type III (1.5 mg mL^−1^) at 37 °C with agitation for 30 min in DMEM containing 10% FBS. Single cell suspensions were obtained by filtration through a 40 µm filter. Tumor cells were isolated from xenografts using the CD326 (EpCAM) Tumor Cell Enrichment and Detection Kit (Cat# 130‐090‐500, Miltenyi Biotec) before ALDH1 staining.

All mice were kept in biosafety level laboratories at the Animal Experiment Center of Sun Yat‐Sen University.

### PET/CT Imaging

The therapeutic efficacy of docetaxel treatment on xenografts was detected by ^18^F‐flurodeoxyglucose (^18^F‐FDG) PET/CT, as previously described.^[^ ^84^ ^]^ After anesthesia, mice were injected with 5 µCi g^−1 18^F‐FDG in 100 µL saline via the tail vein. Forty minutes later, a 15‐minute static scan was performed with an Inveon microPET/CT scanner (Siemens, Germany). Tumor reduction was quantified using the indicator, the percentage injected dose per gram tissue (% ID g^−1^), as previously described.^[^ ^84^ ^]^ Normalized data of each mouse were measured by calculating the ratio of the tumor % ID g^−1^ to the triceps muscle % ID g^−1^.

### TUNEL Assay

The fixed xenografts were processed into 4‐µm‐thick paraffin sections. For the TUNEL assays, the sections were stained using the In Situ Cell Death Detection Kit, TMR red (Cat# 12 156 792 910, Roche) according to the manufacturer's instructions. Sections were then costained with mouse‐anti‐human pan cytokeratin (Cat# ab756, Abcam; 1:100) and subsequently with Alexa Fluor‐conjugated secondary antibodies (Cat# A31571, Thermo Fisher Scientific) for 1 h at 26 °C. DAPI was used to label the nuclei of cells, and images were obtained using a LSM780 confocal microscope (Carl Zeiss).

### NF‐*κ*B Activity Assay

Nuclear extracts were obtained from cell pellets using a Nuclear Extraction Kit (Cat#10 009 277, Cayman Chemical) according to the manufacturer's instructions. Nuclear p65 DNA‐binding activity was determined using an NF‐*κ*B (p65) transcription factor activity assay kit (Cat# 10 007 889, Cayman Chemical) according to the manufacturer's instructions.^[^ ^122^, ^123^ ^]^ Briefly, ELISA plates were pre‐coated with double strand DNA, which was composed of the NF‐*κ*B response element. Nuclear proteins were incubated (5 µg per well) in the plates. After washing, the plates were incubated with primary p65 antibody for 1 h at 26 °C, and the wells were washed with washing buffer. Goat anti‐rabbit horseradish peroxidase‐conjugated secondary antibody was added and p65 activity was detected using a spectrophotometer (readout at 450 nm).

### Statistical Analyses

Statistical tests were performed as indicated in the figure legends. Statistical analyses were performed using GraphPad Prism, with a minimum of three biologically independent samples for significance. Student's *t*‐test was used for comparisons between two groups, and all *t*‐tests were unpaired and two‐tailed. One‐way ANOVA with Tukey's post‐hoc test was used for comparisons among three or more groups. Data are presented as the mean ± SEM. Statistical significance was set at *P* < 0.05, as annotated in legends.

### Ethical Approval for This Study

Informed consent was obtained from each patient and healthy volunteer. Experiments related to samples from breast cancer patients or healthy donors were approved by the Internal Review and Ethics Committees of Sun Yat‐Sen Memorial Hospital (Project No. 2016‐BA‐025). Procedures related to experimental animals were authorized by both ethics boards and the Animal Care and Use Committee of Sun Yat‐Sen University (Project No. L102012019040P).

## Conflict of Interest

The authors declare no conflict of interest.

## Author Contributions

S.Y., Y.L., and A.S. contributed equally to this work. S.S. conceived the ideas, designed the experiments, and wrote the manuscript. S.Y. and Y.W.L. performed the experiments and wrote the manuscript. A. S., J. C., J. L., B. Z., X. L., Q. X., Y. H. L., Q.Z., J. Q. L., M. H., Y. Y., and X. Y. performed the experiments. S.Y., Y.W.L., A. S., S.J., X.W., C.D., J.P., and Q.Z. analyzed the data.

## Supporting information

Supporting Information

## Data Availability

Raw RNA sequencing data have been uploaded to the GEO database (accession number: GSE156470). Peptidomics data have been uploaded to the ProteomeXchange Consortium. The identifiers were PXD021590 and 10.6019/PXD021590.

## References

[1] R. Kalluri , Nat. Rev. Cancer. 2016, 16, 582. 27550820 10.1038/nrc.2016.73

[2] E. Sahai , I. Astsaturov , E. Cukierman , D. G. DeNardo , M. Egeblad , R. M. Evans , D. Fearon , F. R. Greten , S. R. Hingorani , T. Hunter , R. O. Hynes , R. K. Jain , T. Janowitz , C. Jorgensen , A. C. Kimmelman , M. G. Kolonin , R. G. Maki , R. S. Powers , E. Pure , D. C. Ramirez , R. Scherz‐Shouval , M. H. Sherman , S. Stewart , T. D. Tlsty , D. A. Tuveson , F. M. Watt , V. Weaver , A. T. Weeraratna , Z. Werb , Nat. Rev. Cancer. 2020, 20, 174. 31980749 10.1038/s41568-019-0238-1 PMC7046529

[3] V. S. LeBleu , R. Kalluri , Dis. Models Mech. 2018, 11, dmm029447. 10.1242/dmm.029447 PMC5963854 29686035

[4] J. W. Purcell , S. G. Tanlimco , J. Hickson , M. Fox , M. Sho , L. Durkin , T. Uziel , R. Powers , K. Foster , T. McGonigal , S. Kumar , J. Samayoa , D. Zhang , J. P. Palma , S. Mishra , D. Hollenbaugh , K. Gish , S. E. Morgan‐Lappe , E. D. Hsi , D. T. Chao , Cancer Res. 2018, 78, 4059. 29764866 10.1158/0008-5472.CAN-18-0327

[5] K. Narra , S. R. Mullins , H. O. Lee , B. Strzemkowski‐Brun , K. Magalong , V. J. Christiansen , P. A. McKee , B. Egleston , S. J. Cohen , L. M. Weiner , N. J. Meropol , J. D. Cheng , Cancer Biol. Ther. 2007, 6, 1691. 18032930 10.4161/cbt.6.11.4874

[6] H. Sugimoto , T. M. Mundel , M. W. Kieran , R. Kalluri , Cancer Biol. Ther. 2006, 5, 1640. 17106243 10.4161/cbt.5.12.3354

[7] B. C. Ozdemir , T. Pentcheva‐Hoang , J. L. Carstens , X. Zheng , C. C. Wu , T. R. Simpson , H. Laklai , H. Sugimoto , C. Kahlert , S. V. Novitskiy , A. De Jesus‐Acosta , P. Sharma , P. Heidari , U. Mahmood , L. Chin , H. L. Moses , V. M. Weaver , A. Maitra , J. P. Allison , V. S. LeBleu , R. Kalluri , Cancer Cell 2014, 25, 719. 24856586 10.1016/j.ccr.2014.04.005 PMC4180632

[8] A. Costa , Y. Kieffer , A. Scholer‐Dahirel , F. Pelon , B. Bourachot , M. Cardon , P. Sirven , I. Magagna , L. Fuhrmann , C. Bernard , C. Bonneau , M. Kondratova , I. Kuperstein , A. Zinovyev , A. M. Givel , M. C. Parrini , V. Soumelis , A. Vincent‐Salomon , F. Mechta‐Grigoriou , Cancer Cell 2018, 33, 463. 29455927 10.1016/j.ccell.2018.01.011

[9] D. Ohlund , A. Handly‐Santana , G. Biffi , E. Elyada , A. S. Almeida , M. Ponz‐Sarvise , V. Corbo , T. E. Oni , S. A. Hearn , E. J. Lee , Chio II , C. I. Hwang , H. Tiriac , L. A. Baker , D. D. Engle , C. Feig , A. Kultti , M. Egeblad , D. T. Fearon , J. M. Crawford , H. Clevers , Y. Park , D. A. Tuveson , J. Exp. Med. 2017, 214, 579. 28232471 10.1084/jem.20162024 PMC5339682

[10] R. M. Barnett , E. Vilar , J. Natl. Cancer Inst. 2018, 110. 10.1093/jnci/djx131 28922783

[11] H. Korkaya , S. Liu , M. S. Wicha , J. Clin. Invest. 2011, 121, 3804. 21965337 10.1172/JCI57099 PMC3223613

[12] M. E. Sehl , M. S. Wicha , Methods Mol. Biol. 2018, 1711, 333. 29344897 10.1007/978-1-4939-7493-1_16 PMC6322404

[13] T. Oskarsson , E. Batlle , J. Massague , Cell Stem Cell 2014, 14, 306. 24607405 10.1016/j.stem.2014.02.002 PMC3998185

[14] M. Luo , S. G. Clouthier , Y. Deol , S. Liu , S. Nagrath , E. Azizi , M. S. Wicha , Methods Mol. Biol. 2015, 1293, 1. 26040679 10.1007/978-1-4939-2519-3_1

[15] P. Shi , W. Liu , Tala , H. Wang , F. Li , H. Zhang , Y. Wu , Y. Kong , Z. Zhou , C. Wang , W. Chen , R. Liu , C. Chen , Cell Discov. 2017, 3, 17010. 28480051 10.1038/celldisc.2017.10 PMC5396048

[16] J. E. Visvader , G. J. Lindeman , Cell Stem Cell. 2012, 10, 717. 22704512 10.1016/j.stem.2012.05.007

[17] E. Batlle , H. Clevers , Nat. Med. 2017, 23, 1124. 28985214 10.1038/nm.4409

[18] J. Huang , W. Hu , L. Hu , R. A. Previs , H. J. Dalton , X. Y. Yang , Y. Sun , M. McGuire , R. Rupaimoole , A. S. Nagaraja , Y. Kang , T. Liu , A. M. Nick , N. B. Jennings , R. L. Coleman , R. B. Jaffe , A. K. Sood , Mol. Cancer Ther. 2016, 15, 1344. 27009216 10.1158/1535-7163.MCT-15-0144 PMC4893925

[19] K. M. Morgan , B. S. Fischer , F. Y. Lee , J. J. Shah , J. R. Bertino , J. Rosenfeld , A. Singh , H. Khiabanian , S. R. Pine , Mol. Cancer Ther. 2017, 16, 2759. 28978720 10.1158/1535-7163.MCT-17-0439 PMC5716926

[20] J. B. Samon , M. Castillo‐Martin , M. Hadler , A. Ambesi‐Impiobato , E. Paietta , J. Racevskis , P. H. Wiernik , J. M. Rowe , J. Jakubczak , S. Randolph , C. Cordon‐Cardo , A. A. Ferrando , Mol. Cancer Ther. 2012, 11, 1565. 22504949 10.1158/1535-7163.MCT-11-0938 PMC3392513

[21] V. Sosa Iglesias , J. Theys , A. J. Groot , L. M. O. Barbeau , A. Lemmens , A. Yaromina , M. Losen , R. Houben , L. Dubois , M. Vooijs , Front Oncol. 2018, 8, 460. 30464927 10.3389/fonc.2018.00460 PMC6234899

[22] V. Plaks , N. Kong , Z. Werb , Cell Stem Cell. 2015, 16, 225. 25748930 10.1016/j.stem.2015.02.015 PMC4355577

[23] L. Vermeulen , E. M. F. De Sousa , M. van der Heijden , K. Cameron , J. H. de Jong , T. Borovski , J. B. Tuynman , M. Todaro , C. Merz , H. Rodermond , M. R. Sprick , K. Kemper , D. J. Richel , G. Stassi , J. P. Medema , Nat. Cell Biol. 2010, 12, 468. 20418870 10.1038/ncb2048

[24] S. Faivre , G. Demetri , W. Sargent , E. Raymond , Nat. Rev. Drug Discovery 2007, 6, 734. 17690708 10.1038/nrd2380

[25] S. Su , J. Chen , H. Yao , J. Liu , S. Yu , L. Lao , M. Wang , M. Luo , Y. Xing , F. Chen , D. Huang , J. Zhao , L. Yang , D. Liao , F. Su , M. Li , Q. Liu , E. Song , Cell. 2018, 172, 841. 29395328 10.1016/j.cell.2018.01.009

[26] N. Borscheri , A. Roessner , C. Rocken , Am. J. Surg. Pathol. 2001, 25, 1297. 11688465 10.1097/00000478-200110000-00011

[27] K. A. King , J. Hua , J. S. Torday , J. M. Drazen , S. A. Graham , M. A. Shipp , M. E. Sunday , J. Clin. Invest. 1993, 91, 1969. 8486767 10.1172/JCI116417 PMC288193

[28] L. Xie , M. Takahara , T. Nakahara , J. Oba , H. Uchi , S. Takeuchi , Y. Moroi , M. Furue , Arch. Dermatol. Res. 2011, 303, 49. 21076839 10.1007/s00403-010-1093-9

[29] D. Kletsas , E. Caselgrandi , D. Barbieri , D. Stathakos , C. Franceschi , E. Ottaviani , Mech. Ageing Dev. 1998, 102, 15. 9663788 10.1016/s0047-6374(98)00003-7

[30] I. Zagen , P. McLaughlin , J. Neurol. Neurophysiol. 2014, s12.25705564

[31] T. Fukusumi , H. Ishii , M. Konno , T. Yasui , S. Nakahara , Y. Takenaka , Y. Yamamoto , S. Nishikawa , Y. Kano , H. Ogawa , S. Hasegawa , A. Hamabe , N. Haraguchi , Y. Doki , M. Mori , H. Inohara , Br. J. Cancer. 2014, 111, 506. 24874475 10.1038/bjc.2014.289 PMC4119971

[32] Y. Wang , Q. Li , L. Xu , J. Chen , Y. Pu , L. Wang , H. Sun , Y. Guo , C. Guo , OralDis. 2020.

[33] F. Bocci , L. Gearhart‐Serna , M. Boareto , M. Ribeiro , E. Ben‐Jacob , G. R. Devi , H. Levine , J. N. Onuchic , M. K. Jolly , Proc. Natl. Acad. Sci. USA 2019, 116, 148. 30587589 10.1073/pnas.1815345116 PMC6320545

[34] B. P. Roques , F. Noble , V. Dauge , M. C. Fournie‐Zaluski , A. Beaumont , Pharmacol. Rev. 1993, 45, 87. 8475170

[35] L. Mattii , B. Battolla , S. Moscato , R. Fazzi , S. Galimberti , N. Bernardini , A. Dolfi , M. Petrini , Med. Sci. Monit. 2008, 14, BR103. 18509267

[36] B. Battolla , N. Bernardini , M. Petrini , L. Mattii , Med. Sci. Monit. 2011, 17, SC1. 21169922 10.12659/MSM.881309 PMC3524689

[37] Z. Chen , X. Wang , Y. Shao , D. Shi , T. Chen , D. Cui , X. Jiang , Mol. Cell. Biochem. 2011, 358, 221. 21739156 10.1007/s11010-011-0938-7

[38] R. Fazzi , S. Galimberti , R. Testi , S. Pacini , S. Trasciatti , S. Rosini , M. Petrini , Leuk. Res. 2002, 26, 839. 12127560 10.1016/s0145-2126(02)00008-5

[39] Z. Hui , L. Yu , Y. Xiaoli , H. Xiang , Z. Fan , H. Ningbo , Y. Zhigang , L. Ping , Z. Yanhong , M. Qingjun , J. Cell. Biochem. 2007, 101, 1423. 17372927 10.1002/jcb.21258

[40] L. Vanella , D. H. Kim , D. Asprinio , S. J. Peterson , I. Barbagallo , A. Vanella , D. Goldstein , S. Ikehara , A. Kappas , N. G. Abraham , Bone 2010, 46, 236. 19853072 10.1016/j.bone.2009.10.012 PMC2818489

[41] Y. C. Chen , A. Muhlrad , A. Shteyer , M. Vidson , I. Bab , M. Chorev , J. Med. Chem. 2002, 45, 1624. 11931616 10.1021/jm010479l

[42] Z. Greenberg , M. Chorev , A. Muhlrad , A. Shteyer , M. Namdar , N. Mansur , I. Bab , Biochim. Biophys. Acta 1993, 1178, 273. 8364043 10.1016/0167-4889(93)90204-3

[43] I. K. Guttilla , K. N. Phoenix , X. Hong , J. S. Tirnauer , K. P. Claffey , B. A. White , Breast Cancer Res. Treat. 2012, 132, 75. 21553120 10.1007/s10549-011-1534-y

[44] S. C. Su , Q. Liu , J. Q. Chen , J. N. Chen , F. Chen , C. H. He , D. Huang , W. Wu , L. Lin , W. Huang , J. Zhang , X. Y. Cui , F. Zheng , H. Y. Li , H. R. Yao , F. X. Su , E. W. Song , Cancer Cell 2014, 25, 605. 24823638 10.1016/j.ccr.2014.03.021

[45] I. Bab , H. Gavish , M. Namdar‐Attar , A. Muhlrad , Z. Greenberg , Y. Chen , N. Mansur , A. Shteyer , M. Chorev , J. Pept. Res. 1999, 54, 408. 10563506 10.1034/j.1399-3011.1999.00135.x

[46] Z. Greenberg , M. Chorev , A. Muhlrad , A. Shteyer , M. Namdar‐Attar , N. Casap , A. Tartakovsky , M. Vidson , I. Bab , J. Clin. Endocrinol. Metab. 1995, 80, 2330. 7629225 10.1210/jcem.80.8.7629225

[47] I. Bab , E. Smith , H. Gavish , M. Attar‐Namdar , M. Chorev , Y. C. Chen , A. Muhlrad , M. J. Birnbaum , G. Stein , B. Frenkel , J. Biol. Chem. 1999, 274, 14474. 10318873 10.1074/jbc.274.20.14474

[48] M. M. Gaelzer , M. S. D. Santos , B. P. Coelho , A. H. de Quadros , F. Simao , V. Usach , F. C. R. Guma , P. Setton‐Avruj , G. Lenz , C. G. Salbego , Mol. Neurobiol. 2017, 54, 6261. 27714633 10.1007/s12035-016-0126-6

[49] I. Bab , M. Chorev , Biopolymers 2002, 66, 33. 12228919 10.1002/bip.10202

[50] T. J. Rettenmaier , J. D. Sadowsky , N. D. Thomsen , S. C. Chen , A. K. Doak , M. R. Arkin , J. A. Wells , Proc. Natl. Acad. Sci. USA 2014, 111, 18590. 25518860 10.1073/pnas.1415365112 PMC4284534

[51] Y. C. Chen , I. Bab , N. Mansur , A. Muhlrad , A. Shteyer , M. Namdar‐Attar , H. Gavish , M. Vidson , M. Chorev , J. Pept. Res. 2000, 56, 147. 11007271 10.1034/j.1399-3011.2000.00763.x

[52] P. C. Bailey , R. M. Lee , M. I. Vitolo , S. J. P. Pratt , E. Ory , K. Chakrabarti , C. J. Lee , K. N. Thompson , S. S. Martin , iScience 2018, 8, 29. 30268511 10.1016/j.isci.2018.08.015 PMC6170521

[53] A. Cicalese , G. Bonizzi , C. E. Pasi , M. Faretta , S. Ronzoni , B. Giulini , C. Brisken , S. Minucci , P. P. Di Fiore , P. G. Pelicci , Cell 2009, 138, 1083. 19766563 10.1016/j.cell.2009.06.048

[54] P. Ji , Y. Zhang , S. J. Wang , H. L. Ge , G. P. Zhao , Y. C. Xu , Y. Wang , Oncol. Rep. 2016, 35, 3293. 27109463 10.3892/or.2016.4739

[55] J. Li , S. Condello , J. Thomes‐Pepin , X. Ma , Y. Xia , T. D. Hurley , D. Matei , J. X. Cheng , Cell Stem Cell 2017, 20, 303. 28041894 10.1016/j.stem.2016.11.004 PMC5337165

[56] O. Yanes , J. Clark , D. M. Wong , G. J. Patti , A. Sanchez‐Ruiz , H. P. Benton , S. A. Trauger , C. Desponts , S. Ding , G. Siuzdak , Nat. Chem. Biol. 2010, 6, 411. 20436487 10.1038/nchembio.364 PMC2873061

[57] X. Y. Qin , T. Su , W. Yu , S. Kojima , Cell Death Dis. 2020, 11, 66. 31988297 10.1038/s41419-020-2257-y PMC6985230

[58] J. Rios‐Esteves , M. D. Resh , Cell Rep. 2013, 4, 1072. 24055053 10.1016/j.celrep.2013.08.027 PMC3845236

[59] A. Parrales , A. Ranjan , T. Iwakuma , Stem Cell Invest. 2017, 4, 49. 10.21037/sci.2017.05.07 PMC5460094 28607923

[60] D. Mauvoisin , G. Rocque , O. Arfa , A. Radenne , P. Boissier , C. Mounier , J. Cell Commun. Signaling 2007, 1, 113. 10.1007/s12079-007-0011-1 PMC2275876 18481202

[61] D. Luyimbazi , A. Akcakanat , P. F. McAuliffe , L. Zhang , G. Singh , A. M. Gonzalez‐Angulo , H. Chen , K. A. Do , Y. Zheng , M. C. Hung , G. B. Mills , F. Meric‐Bernstam , Mol. Cancer Ther. 2010, 9, 2770. 20876744 10.1158/1535-7163.MCT-09-0980 PMC2965451

[62] H. Kim , C. Rodriguez‐Navas , R. K. Kollipara , P. Kapur , I. Pedrosa , J. Brugarolas , R. Kittler , J. Ye , Cell Rep. 2015, 13, 495. 26456834 10.1016/j.celrep.2015.09.010 PMC4618234

[63] D. P. Bagchi , A. Nishii , Z. Li , J. B. DelProposto , C. A. Corsa , H. Mori , J. Hardij , B. S. Learman , C. N. Lumeng , O. A. MacDougald , Mol. Metab. 2020, 42, 101078. 32919095 10.1016/j.molmet.2020.101078 PMC7554252

[64] M. A. Huber , N. Azoitei , B. Baumann , S. Grunert , A. Sommer , H. Pehamberger , N. Kraut , H. Beug , T. Wirth , J. Clin. Invest. 2004, 114, 569. 15314694 10.1172/JCI21358 PMC503772

[65] N. Erez , M. Truitt , P. Olson , S. T. Arron , D. Hanahan , Cancer Cell. 2010, 17, 135. 20138012 10.1016/j.ccr.2009.12.041

[66] D. V. Catenacci , M. R. Junttila , T. Karrison , N. Bahary , M. N. Horiba , S. R. Nattam , R. Marsh , J. Wallace , M. Kozloff , L. Rajdev , D. Cohen , J. Wade , B. Sleckman , H. J. Lenz , P. Stiff , P. Kumar , P. Xu , L. Henderson , N. Takebe , R. Salgia , X. Wang , W. M. Stadler , F. J. de Sauvage , H. L. Kindler , J. Clin. Oncol. 2015, 33, 4284. 26527777 10.1200/JCO.2015.62.8719 PMC4678179

[67] R. D. Hofheinz , S. E. al‐Batran , F. Hartmann , G. Hartung , D. Jager , C. Renner , P. Tanswell , U. Kunz , A. Amelsberg , H. Kuthan , G. Stehle , Onkologie 2003, 26, 44. 12624517 10.1159/000069863

[68] C. K. Pallangyo , P. K. Ziegler , F. R. Greten , J. Exp. Med. 2015, 212, 2253. 26621452 10.1084/jem.20150576 PMC4689166

[69] C. Feig , J. O. Jones , M. Kraman , R. J. Wells , A. Deonarine , D. S. Chan , C. M. Connell , E. W. Roberts , Q. Zhao , O. L. Caballero , S. A. Teichmann , T. Janowitz , D. I. Jodrell , D. A. Tuveson , D. T. Fearon , Proc. Natl. Acad. Sci. USA 2013, 110, 20212. 24277834 10.1073/pnas.1320318110 PMC3864274

[70] A. Orimo , P. B. Gupta , D. C. Sgroi , F. Arenzana‐Seisdedos , T. Delaunay , R. Naeem , V. J. Carey , A. L. Richardson , R. A. Weinberg , Cell 2005, 121, 335. 15882617 10.1016/j.cell.2005.02.034

[71] A. Calon , E. Espinet , S. Palomo‐Ponce , D. V. Tauriello , M. Iglesias , M. V. Cespedes , M. Sevillano , C. Nadal , P. Jung , X. H. Zhang , D. Byrom , A. Riera , D. Rossell , R. Mangues , J. Massague , E. Sancho , E. Batlle , Cancer Cell. 2012, 22, 571. 23153532 10.1016/j.ccr.2012.08.013 PMC3512565

[72] M. Yu , G. Guo , L. Huang , L. Deng , C. S. Chang , B. R. Achyut , M. Canning , N. Xu , A. S. Arbab , R. J. Bollag , P. C. Rodriguez , A. L. Mellor , H. Shi , D. H. Munn , Y. Cui , Nat. Commun. 2020, 11, 515. 31980601 10.1038/s41467-019-14060-x PMC6981126

[73] M. A. Lakins , E. Ghorani , H. Munir , C. P. Martins , J. D. Shields , Nat. Commun. 2018, 9, 948. 29507342 10.1038/s41467-018-03347-0 PMC5838096

[74] I. V. Pinchuk , J. I. Saada , E. J. Beswick , G. Boya , S. M. Qiu , R. C. Mifflin , G. S. Raju , V. E. Reyes , D. W. Powell , Gastroenterology 2008, 135, 1228. 18760278 10.1053/j.gastro.2008.07.016 PMC2584612

[75] A. J. Barrett , N. D. Rawlings , J. Woessner , Handbook of Proteolytic Enzymes, Academic Press, San Diego, CA 1998.

[76] A. F. Ruchon , M. Marcinkiewicz , K. Ellefsen , A. Basak , J. Aubin , P. Crine , G. Boileau , J. Bone Miner. Res. 2000, 15, 1266. 10893675 10.1359/jbmr.2000.15.7.1266

[77] A. M. Intlekofer , L. W. S. Finley , Nat. Metab. 2019, 1, 177. 31245788 10.1038/s42255-019-0032-0 PMC6594714

[78] C. D. Folmes , S. Park , A. Terzic , Cell Metab. 2013, 17, 153. 23395162 10.1016/j.cmet.2013.01.010

[79] H. Liang , J. Xiao , Z. Zhou , J. Wu , F. Ge , Z. Li , H. Zhang , J. Sun , F. Li , R. Liu , C. Chen , Oncogene 2018, 37, 1961. 29367761 10.1038/s41388-017-0089-8 PMC5895606

[80] H. Sampath , J. M. Ntambi , Future Lipidol. 2008, 3, 163.

[81] Z. X. Chen , M. Chang , Y. L. Peng , L. Zhao , Y. R. Zhan , L. J. Wang , R. Wang , Regul. Pept. 2007, 142, 16. 17331598 10.1016/j.regpep.2007.01.003

[82] S. C. Pigossi , M. C. Medeiros , S. Saska , J. A. Cirelli , R. M. Scarel‐Caminaga , Int. J. Mol. Sci. 2016, 17, 1885. 10.3390/ijms17111885 PMC5133884 27879684

[83] G. M. Policastro , M. L. Becker , Wiley Interdiscip. Rev.: Nanomed. Nanobiotechnol. 2016, 8, 449. 26391307 10.1002/wnan.1376

[84] Y. Lu , Q. Zhao , J. Y. Liao , E. Song , Q. Xia , J. Pan , Y. Li , J. Li , B. Zhou , Y. Ye , C. Di , S. Yu , Y. Zeng , S. Su , Cell 2020, 180, 1081. 32142650 10.1016/j.cell.2020.02.015

[85] S. Saska , R. M. Scarel‐Caminaga , L. N. Teixeira , L. P. Franchi , R. A. Dos Santos , A. M. Gaspar , P. T. de Oliveira , A. L. Rosa , C. S. Takahashi , Y. Messaddeq , S. J. Ribeiro , R. Marchetto , J. Mater. Sci.: Mater. Med. 2012, 23, 2253. 22622695 10.1007/s10856-012-4676-5

[86] S. Yousefnia , K. Ghaedi , F. Seyed Forootan , M. H. Nasr Esfahani , Tumor Biol. 2019, 41, 101042831986910. 10.1177/1010428319869101 31423948

[87] M. Samoszuk , J. Tan , G. Chorn , Breast Cancer Res. 2005, 7, R274. 15987422 10.1186/bcr995 PMC1143574

[88] G. Dontu , W. M. Abdallah , J. M. Foley , K. W. Jackson , M. F. Clarke , M. J. Kawamura , M. S. Wicha , Genes Dev. 2003, 17, 1253. 12756227 10.1101/gad.1061803 PMC196056

[89] S. Su , Q. Liu , J. Chen , J. Chen , F. Chen , C. He , D. Huang , W. Wu , L. Lin , W. Huang , J. Zhang , X. Cui , F. Zheng , H. Li , H. Yao , F. Su , E. Song , Cancer Cell 2014, 25, 605. 24823638 10.1016/j.ccr.2014.03.021

[90] Z. Greenberg , H. Gavish , A. Muhlrad , M. Chorev , A. Shteyer , M. Attar‐Namdar , A. Tartakovsky , I. Bab , J. Cell. Biochem. 1997, 65, 359. 9138092

[91] C. N. Papandreou , B. Usmani , Y. Geng , T. Bogenrieder , R. Freeman , S. Wilk , C. L. Finstad , V. E. Reuter , C. T. Powell , D. Scheinberg , C. Magill , H. I. Scher , A. P. Albino , D. M. Nanus , Nat. Med. 1998, 4, 50. 9427606 10.1038/nm0198-050

[92] M. A. Shipp , G. E. Tarr , C. Y. Chen , S. N. Switzer , L. B. Hersh , H. Stein , M. E. Sunday , E. L. Reinherz , Proc. Natl. Acad. Sci. USA 1991, 88, 10662. 1660144 10.1073/pnas.88.23.10662 PMC52990

[93] M. Sumitomo , T. Asano , J. Asakuma , T. Asano , D. M. Nanus , M. Hayakawa , Clin. Cancer Res. 2004, 10, 260. 14734478 10.1158/1078-0432.ccr-0798-3

[94] W. L. Hwang , M. H. Yang , Cell Cycle 2016, 15, 2697. 27580100 10.1080/15384101.2016.1218101 PMC5053571

[95] P. H. Petersen , K. Zou , J. K. Hwang , Y. N. Jan , W. Zhong , Nature 2002, 419, 929. 12410312 10.1038/nature01124

[96] Z. Xue , H. Yan , J. Li , S. Liang , X. Cai , X. Chen , Q. Wu , L. Gao , K. Wu , Y. Nie , D. Fan , J. Cell. Biochem. 2012, 113, 302. 21913215 10.1002/jcb.23356

[97] G. M. Lee , S. S. Fong , D. J. Oh , K. Francis , B. O. Palsson , In Vitro Cell. Dev. Biol.: Anim. 2002, 38, 90. 11929001 10.1290/1071-2690(2002)038<0090:CAEOPA>2.0.CO;2

[98] S. Su , J. Zhao , Y. Xing , X. Zhang , J. Liu , Q. Ouyang , J. Chen , F. Su , Q. Liu , E. Song , Cell 2018, 175, 442. 30290143 10.1016/j.cell.2018.09.007

[99] R. Eil , S. K. Vodnala , D. Clever , C. A. Klebanoff , M. Sukumar , J. H. Pan , D. C. Palmer , A. Gros , T. N. Yamamoto , S. J. Patel , G. C. Guittard , Z. Yu , V. Carbonaro , K. Okkenhaug , D. S. Schrump , W. M. Linehan , R. Roychoudhuri , N. P. Restifo , Nature 2016, 537, 539. 27626381 10.1038/nature19364 PMC5204372

[100] H. Haslene‐Hox , E. Oveland , K. C. Berg , O. Kolmannskog , K. Woie , H. B. Salvesen , O. Tenstad , H. Wiig , PLoS One 2011, 6, 19217. 10.1371/journal.pone.0019217 PMC3082557 21541282

[101] P. C. Ho , J. D. Bihuniak , A. N. Macintyre , M. Staron , X. Liu , R. Amezquita , Y. C. Tsui , G. Cui , G. Micevic , J. C. Perales , S. H. Kleinstein , E. D. Abel , K. L. Insogna , S. Feske , J. W. Locasale , M. W. Bosenberg , J. C. Rathmell , S. M. Kaech , Cell 2015, 162, 1217. 26321681 10.1016/j.cell.2015.08.012 PMC4567953

[102] M. Holtta , H. Zetterberg , E. Mirgorodskaya , N. Mattsson , K. Blennow , J. Gobom , PLoS One 2012, 7, 42555. 10.1371/journal.pone.0042555 PMC3412831 22880031

[103] P. Majerova , P. Barath , A. Michalicova , S. Katina , M. Novak , A. Kovac , J Alzheimer's Dis. 2017, 58, 507. 28453489 10.3233/JAD-170110

[104] J. Wang , R. Cunningham , H. Zetterberg , S. Asthana , C. Carlsson , O. Okonkwo , L. Li , Proteomics: Clin Appl. 2016, 10, 1225. 27863112 10.1002/prca.201600009

[105] G. Sathe , C. H. Na , S. Renuse , A. K. Madugundu , M. Albert , A. Moghekar , A. Pandey , Proteomics: Clin Appl. 2019, 13, 1800105. 10.1002/prca.201800105 PMC6639119 30578620

[106] B. L. Parker , J. G. Burchfield , D. Clayton , T. A. Geddes , R. J. Payne , B. Kiens , J. F. P. Wojtaszewski , E. A. Richter , D. E. James , Mol. Cell. Proteomics 2017, 16, 2055. 28982716 10.1074/mcp.RA117.000020 PMC5724171

[107] A. Devabhaktuni , S. Lin , L. Zhang , K. Swaminathan , C. G. Gonzalez , N. Olsson , S. M. Pearlman , K. Rawson , J. E. Elias , Nat. Biotechnol. 2019, 37, 469. 30936560 10.1038/s41587-019-0067-5 PMC6447449

[108] E. W. Deutsch , N. Bandeira , V. Sharma , Y. Perez‐Riverol , J. J. Carver , D. J. Kundu , D. Garcia‐Seisdedos , A. F. Jarnuczak , S. Hewapathirana , B. S. Pullman , J. Wertz , Z. Sun , S. Kawano , S. Okuda , Y. Watanabe , H. Hermjakob , B. MacLean , M. J. MacCoss , Y. Zhu , Y. Ishihama , J. A. Vizcaino , Nucleic Acids Res. 2020, 48, D1145. 31686107 10.1093/nar/gkz984 PMC7145525

[109] K. M. Carvalho , G. Boileau , A. C. Camargo , L. Juliano , Anal. Biochem. 1996, 237, 167. 8660561 10.1006/abio.1996.0224

[110] O. B. Goodman Jr. , M. Febbraio , R. Simantov , R. Zheng , R. Shen , R. L. Silverstein , D. M. Nanus , J. Biol. Chem. 2006, 281, 33597. 16940054 10.1074/jbc.M602490200

[111] N. Parmentier , V. Stroobant , D. Colau , P. de Diesbach , S. Morel , J. Chapiro , P. van Endert , B. J. Van den Eynde , Nat. Immunol. 2010, 11, 449. 20364150 10.1038/ni.1862

[112] W. Li , L. Lin , D. Yan , Y. Jin , Y. Xu , Y. Li , M. Ma , Z. Wu , Anal. Chem. 2020, 92, 3237. 31961136 10.1021/acs.analchem.9b05046

[113] F. Madeira , Y. M. Park , J. Lee , N. Buso , T. Gur , N. Madhusoodanan , P. Basutkar , A. R. N. Tivey , S. C. Potter , R. D. Finn , R. Lopez , Nucleic Acids Res. 2019, 47, W636. 30976793 10.1093/nar/gkz268 PMC6602479

[114] S. R. Kumar , T. P. Quinn , S. L. Deutscher , Clin. Cancer Res. 2007, 13, 6070. 17947470 10.1158/1078-0432.CCR-07-0160

[115] N. G. Karasseva , V. V. Glinsky , N. X. Chen , R. Komatireddy , T. P. Quinn , J Protein Chem. 2002, 21, 287. 12168699 10.1023/a:1019749504418

[116] K. Shan , C. Wang , W. Liu , K. Liu , B. Jia , L. Hao , Sci. Data 2019, 6, 10. 30918266 10.1038/s41597-019-0012-y PMC6437646

[117] Q. L. He , X. Y. Wei , X. Y. Han , Q. Zhou , H. Q. Wang , N. Z. Ding , X. Q. Meng , H. Schatten , Q. Y. Sun , S. Z. Liu , Arch. Toxicol. 2019, 93, 2575. 31388691 10.1007/s00204-019-02529-z

[118] S. Ducheix , C. Peres , J. Hardfeldt , C. Frau , G. Mocciaro , E. Piccinin , J. M. Lobaccaro , S. De Santis , M. Chieppa , J. Bertrand‐Michel , M. Plateroti , J. L. Griffin , C. Sabba , J. M. Ntambi , A. Moschetta , Gastroenterology 2018, 155, 1524. 30063922 10.1053/j.gastro.2018.07.032

[119] X. You , W. Jiang , W. Lu , H. Zhang , T. Yu , J. Tian , S. Wen , G. Garcia‐Manero , P. Huang , Y. Hu , Cancer Commun. 2019, 39, 17. 10.1186/s40880-019-0362-z PMC6449955 30947742

[120] H. Zhang , M. G. Badur , A. S. Divakaruni , S. J. Parker , C. Jager , K. Hiller , A. N. Murphy , C. M. Metallo , Cell Rep. 2016, 16, 1536. 27477285 10.1016/j.celrep.2016.06.102 PMC4981511

[121] B. Liu , L. Sun , Q. Liu , C. Gong , Y. Yao , X. Lv , L. Lin , H. Yao , F. Su , D. Li , M. Zeng , E. Song , Cancer Cell 2015, 27, 370. 25759022 10.1016/j.ccell.2015.02.004

[122] M. V. Badiwala , D. Guha , L. Tumiati , J. Joseph , A. Ghashghai , H. J. Ross , D. H. Delgado , V. Rao , Circulation 2011, 124, S197. 21911813 10.1161/CIRCULATIONAHA.110.011734

[123] M. H. Zaki , P. Vogel , R. K. Malireddi , M. Body‐Malapel , P. K. Anand , J. Bertin , D. R. Green , M. Lamkanfi , T. D. Kanneganti , Cancer Cell 2011, 20, 649. 22094258 10.1016/j.ccr.2011.10.022 PMC3761879

[124] K. Wiesehan , K. Buder , R. P. Linke , S. Patt , M. Stoldt , E. Unger , B. Schmitt , E. Bucci , D. Willbold , ChemBioChem 2003, 4, 748. 12898626 10.1002/cbic.200300631

[125] M. Li , C. P. Anastassiades , B. Joshi , C. M. Komarck , C. Piraka , B. J. Elmunzer , D. K. Turgeon , T. D. Johnson , H. Appelman , D. G. Beer , T. D. Wang , Gastroenterology 2010, 139, 1472. 20637198 10.1053/j.gastro.2010.07.007 PMC3319360

[126] D. Knappe , S. Piantavigna , A. Hansen , A. Mechler , A. Binas , O. Nolte , L. L. Martin , R. Hoffmann , J. Med. Chem. 2010, 53, 5240. 20565063 10.1021/jm100378b

[127] M. H. Kim , S. G. Kim , D. W. Kim , Nucl. Med. Mol. Imaging 2018, 52, 359. 30344784 10.1007/s13139-018-0535-8 PMC6177343

[128] L. I. Fedoreyeva , Kireev II , V. Khavinson , B. F. Vanyushin , Biochemistry 2011, 76, 1210. 22117547 10.1134/S0006297911110022

[129] Q. Y. Cai , P. Yu , C. Besch‐Williford , C. J. Smith , G. L. Sieckman , T. J. Hoffman , L. Ma , Prostate 2013, 73, 842. 23280511 10.1002/pros.22630

[130] M. Gijs , G. Penner , G. B. Blackler , N. R. Impens , S. Baatout , A. Luxen , A. M. Aerts , Pharmaceuticals 2016, 9, 29. 10.3390/ph9020029 PMC4932547 27213406

[131] A. Paulus , P. Desai , B. Carney , G. Carlucci , T. Reiner , C. Brand , W. A. Weber , EJNMMI Res. 2015, 5, 120. 26285667 10.1186/s13550-015-0120-4 PMC4540712

