# Supplementary material for: A CD10‐OGP Membrane Peptolytic Signaling Axis in Fibroblasts Regulates Lipid Metabolism of Cancer Stem Cells via SCD1
Source: Adv Sci (Weinh). 2021 Aug 7;8(19):2101848. doi: 10.1002/advs.202101848 (PMC8498877; doi:10.1002/advs.202101848)
Supplement: Supplementary file 1 — Supporting Information [file ADVS-8-2101848-s001.pdf]

© 2021 Wiley-VCH GmbH

ADVANCED SCIENCE
Open Access

Supporting Information

for *Adv. Sci.,* DOI: 10.1002/advs.202101848

A CD10-OGP Membrane Peptolytic Signaling Axis in Fibroblasts Regulates Lipid Metabolism of Cancer Stem Cells via SCD1

Shubin Yu, Yiwen Lu, An Su, Jianing Chen, Jiang Li, Boxuan Zhou, Xinwei Liu, Qidong Xia, Yihong Li, Jiaqian Li, Min Huang, Yingying Ye, Qiyi Zhao, Sushi Jiang, Xiaoqing Yan, Xiaojuan Wang, Can Di, Jiayao Pan, and *Shicheng Su**

Supporting Information

A CD10-OGP Membrane Peptolytic Signaling Axis in Fibroblasts Regulates Lipid Metabolism of Cancer Stem Cells via SCD1

*Shubin Yu†, Yiwen Lu†, An Su†, Jianing Chen, Jiang Li, Boxuan Zhou, Xinwei Liu, Qidong Xia, Yihong Li, Jiaqian Li, Min Huang, Yingying Ye, Qiyi Zhao, Sushi Jiang, Xiaoqing Yan, Xiaojuan Wang, Can Di, Jiayao Pan, Shicheng Su**

†These authors contributed equally to this work.
* Corresponding author.

Supplemental Figure 1

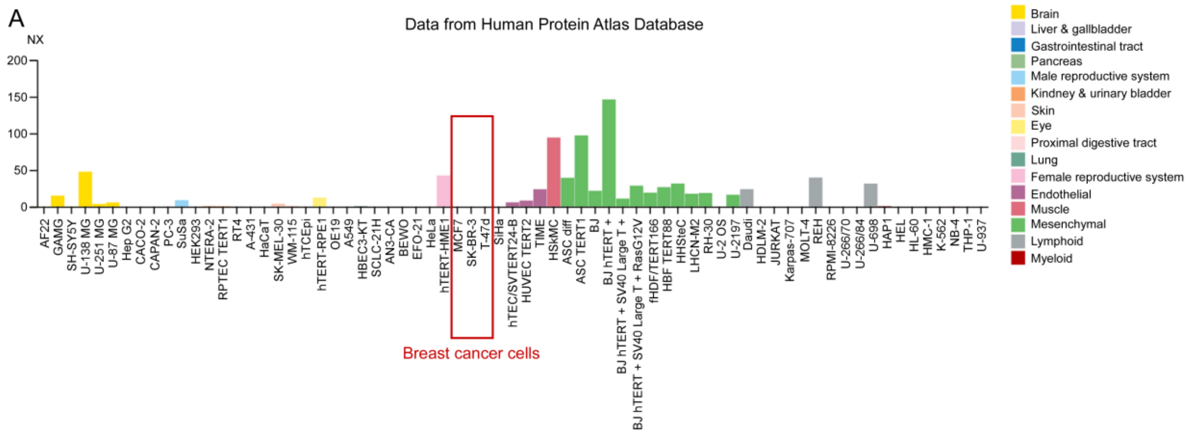

Figure S1 CD10 expression in CAFs

A The CD10 expression profile in multiple cancer cell lines from the Human Protein Atlas database.

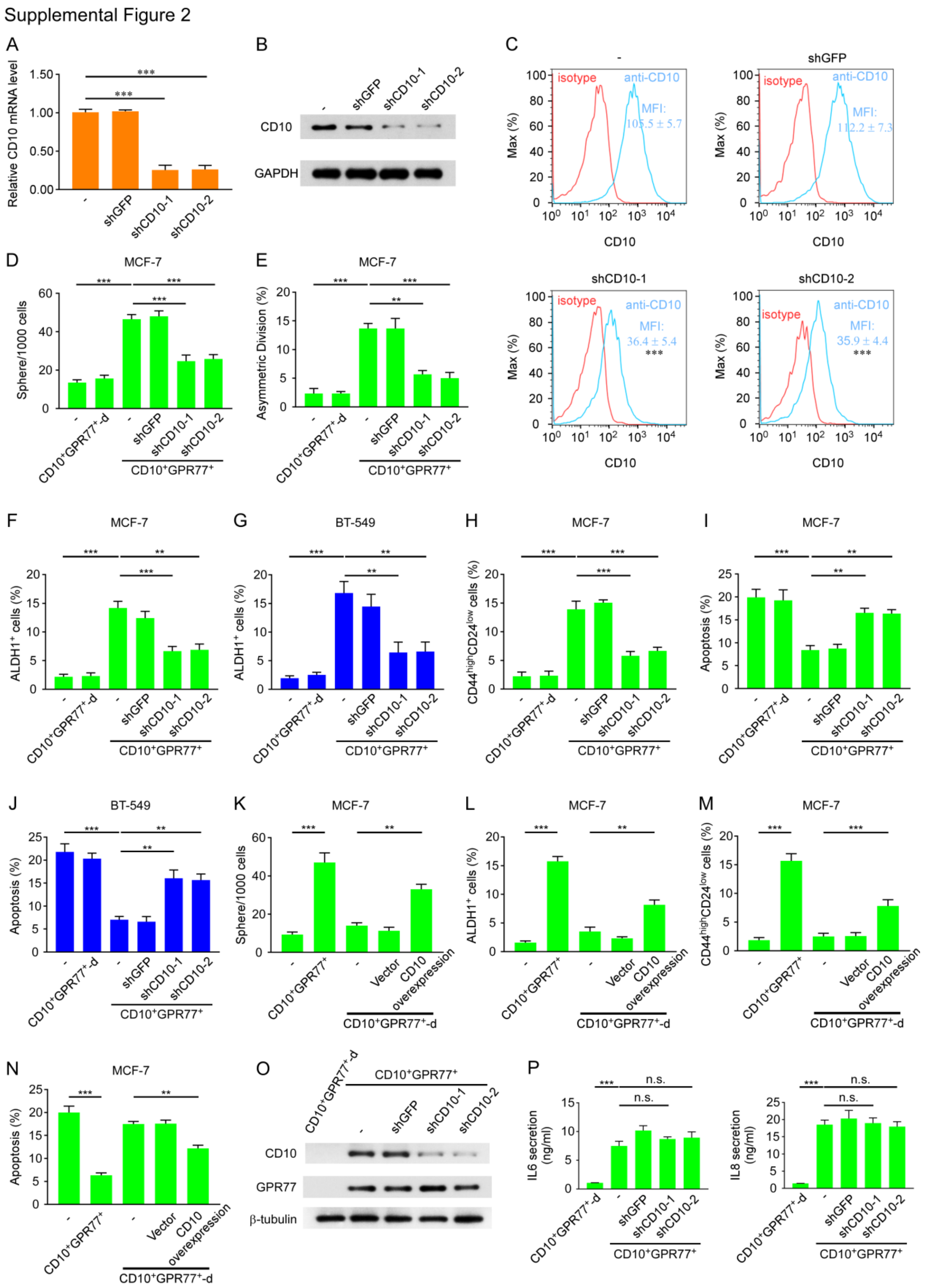

Figure S2. CD10 in CAFs sustains cancer stemness and chemoresistance

A-C $CD10^+GPR77^+$ CAFs were transduced with GFP shRNA or CD10 shRNA.

A Expression of CD10 mRNA detected by qRT-PCR (n = 3).

B CD10 levels were determined by western blotting (n = 3). Representative blots are shown.

C Flow cytometry analysis of CD10 levels. Numerical values indicate mean fluorescence intensity (MFI × 10^2). Mean ± SEM, n = 3. ****P* < 0.001 compared with untreated CAFs by one-way ANOVA.

D Quantification of Figure 2A (n = 6).

E Quantification of Numb staining in Figure 2B (n = 3).

F Quantification of Figure 2C (n = 3).

G Quantification of Figure 2D (n = 3).

H Quantification of Figure 2E (n = 3).

I Quantification of Figure 2F (n = 4).

J Quantification of Figure 2G (n = 3).

K Quantification of Figure 2I (n = 3).

L Quantification of Figure 2J (n = 3).

M Quantification of Figure 2K (n = 3).

N Quantification of Figure 2L (n = 3).

O, P $CD10^+GPR77^+$ CAFs were transduced without (-) or with shGFP or shCD10.

O Representative immunoblots for GPR77 and CD10 in the indicated CAFs (n = 3).

P The levels of IL-6 and IL-8 in the supernatants of the indicated CAFs were determined by ELISA (n = 3).

Mean ± SEM, n.s., not significant; **P* < 0.05; ***P* < 0.01; ****P* < 0.001 by one-way ANOVA.

Supplemental Figure 3

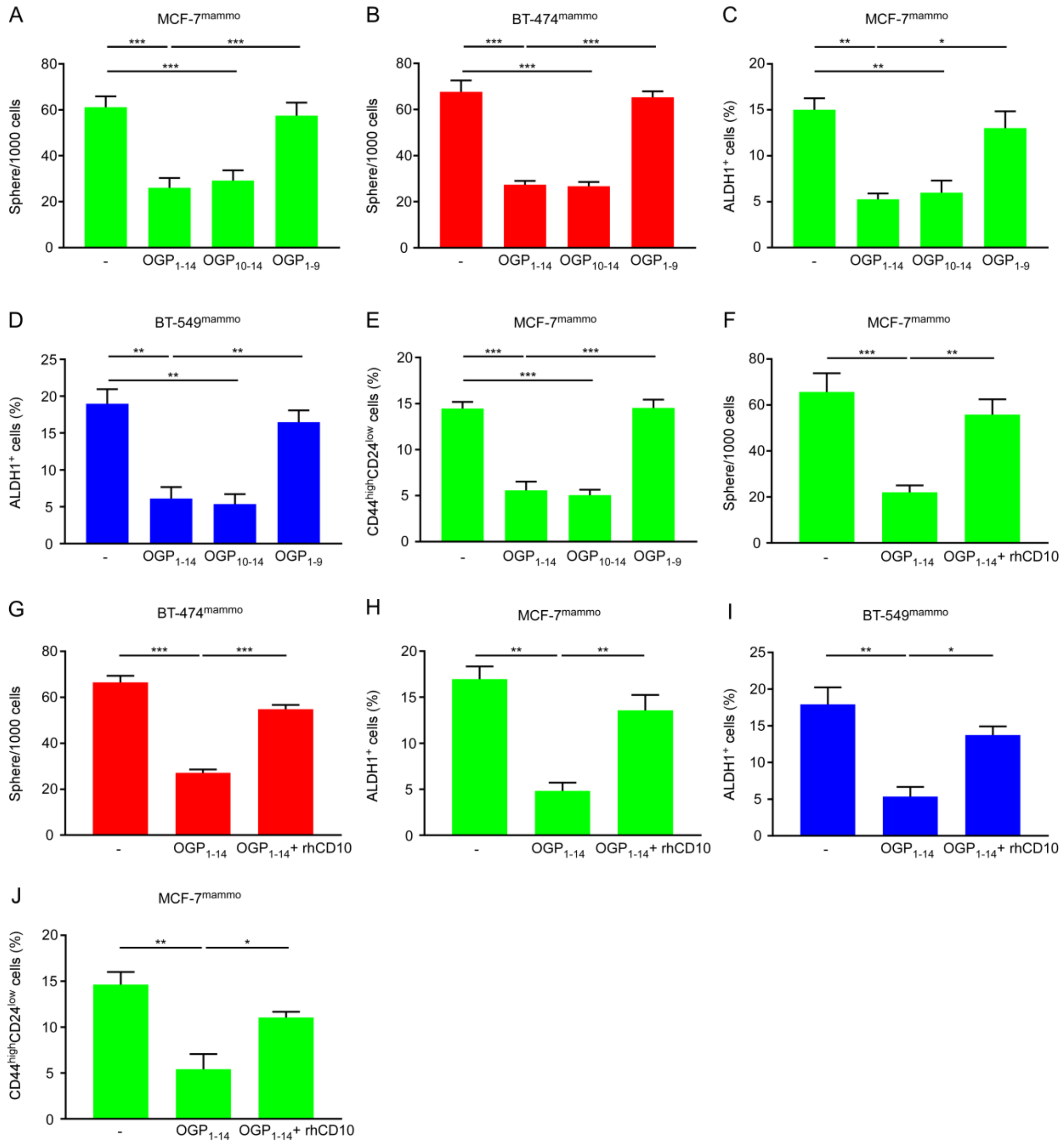

Figure S3. OGP suppresses CSCs via the YGFGG domain that can be cleaved by CD10

A Quantification of Figure 4A (n = 6).

B Quantification of Figure 4B (n = 6).

C Quantification of Figure 4C (n = 3).

D Quantification of Figure 4D (n = 3).

E Quantification of Figure 4E (n = 3).

F Quantification of Figure 4F (n = 6).

G Quantification of Figure 4G (n = 6).

H Quantification of Figure 4H (n = 3).

I Quantification of Figure 4I (n = 3).

J Quantification of Figure 4J (n = 3).

A-J, Mean ± SEM, $*P < 0.05$; $**P < 0.01$; $***P < 0.001$ by one-way ANOVA.

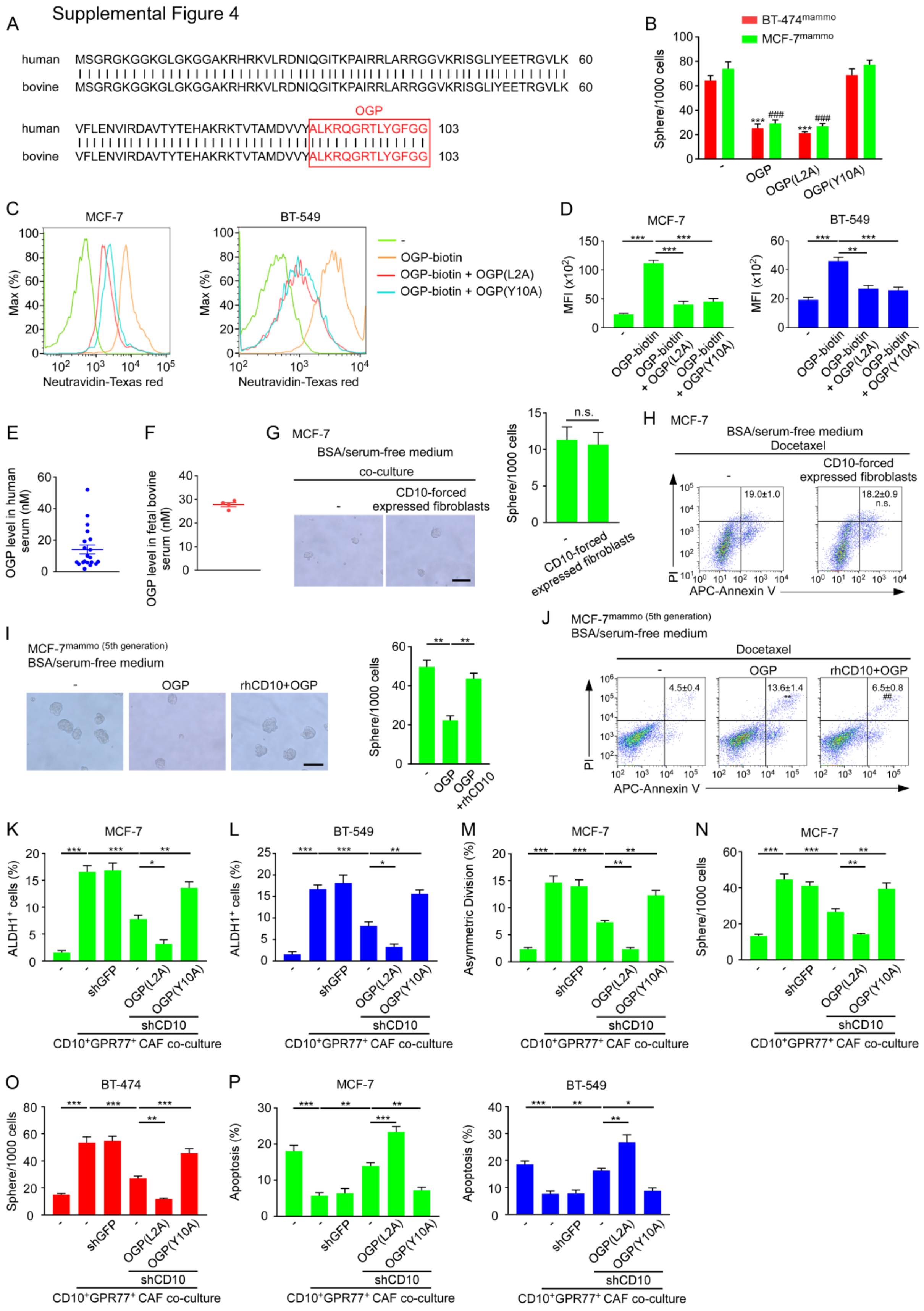

Figure S4. CD10 supports CSCs by cleavage of OGP

A The human and bovine amino acid sequences of histone H4, from which OGP is derived by alternative translational initiation, are identical [1].

B Quantification of Figure 5A, B. Mean ± SEM, n = 6 for each cell line. $^{***}P < 0.001$ and $^{\#\#\#}P < 0.001$ compared with untreated BT-474^{mammo} cells and MCF-7^{mammo} cells by one-way ANOVA, respectively.

C, D Indicated tumor cells were treated as shown in Figure 5C and D. Biotinylated OGP bound to cells was detected by flow cytometry. Representative histograms **(C)** and MFI (×10^2) quantification **(D)** are shown. Mean ± SEM, n = 3. $^{**}P < 0.01$; $^{***}P < 0.001$ by one-way ANOVA.

E OGP levels in human serum were evaluated by ELISA. Mean ± SEM, n = 20.

F OGP levels in fetal bovine serum were evaluated using ELISA. Mean ± SEM, n = 4.

G, H MCF-7 cells were cultured alone or co-cultured with fibroblasts with CD10 forced expression in BSA/serum-free medium.

G Representative images (left) and quantification (right) of mammosphere formation in MCF-7 cells. Scale bar, 100 μm. Mean ± SEM, n=3. n.s., not significant by the Student's t-test.

H After the co-culture, MCF-7 cells were treated with docetaxel. The apoptosis of tumor cells was determined by flow cytometry. Mean ± SEM, n=3. n.s., not significant by the Student's t-test.

I, J MCF-7^{mammo} cells were untreated or treated with OGP in the presence or absence of rhCD10 in serum/BSA-free mammosphere medium.

I Representative images (left) and quantification (right) of mammosphere formation are shown (mean ± SEM, n = 3). $^{**}P < 0.01$ by one-way ANOVA.

J Apoptosis of MCF-7^{mammo} treated with docetaxel was determined by flow cytometry. Mean ± SEM, n = 3. $^{**}P < 0.01$ compared with the untreated group, $^{\#\#}P < 0.01$ compared with the OGP-treatment group by one-way ANOVA.

K Quantification of Figure 5E (n = 4).

L Quantification of Figure 5F (n = 4).

M Quantification of Figure 5H (n = 3).

N Quantification of Figure 5I (n = 6).

O Quantification of Figure 5J (n = 6).

P Quantification of Figure 5K (n = 4 for each cell line).

K-P, Mean ± SEM, **P* < 0.05; ***P* < 0.01; ****P* < 0.001 by one-way ANOVA.

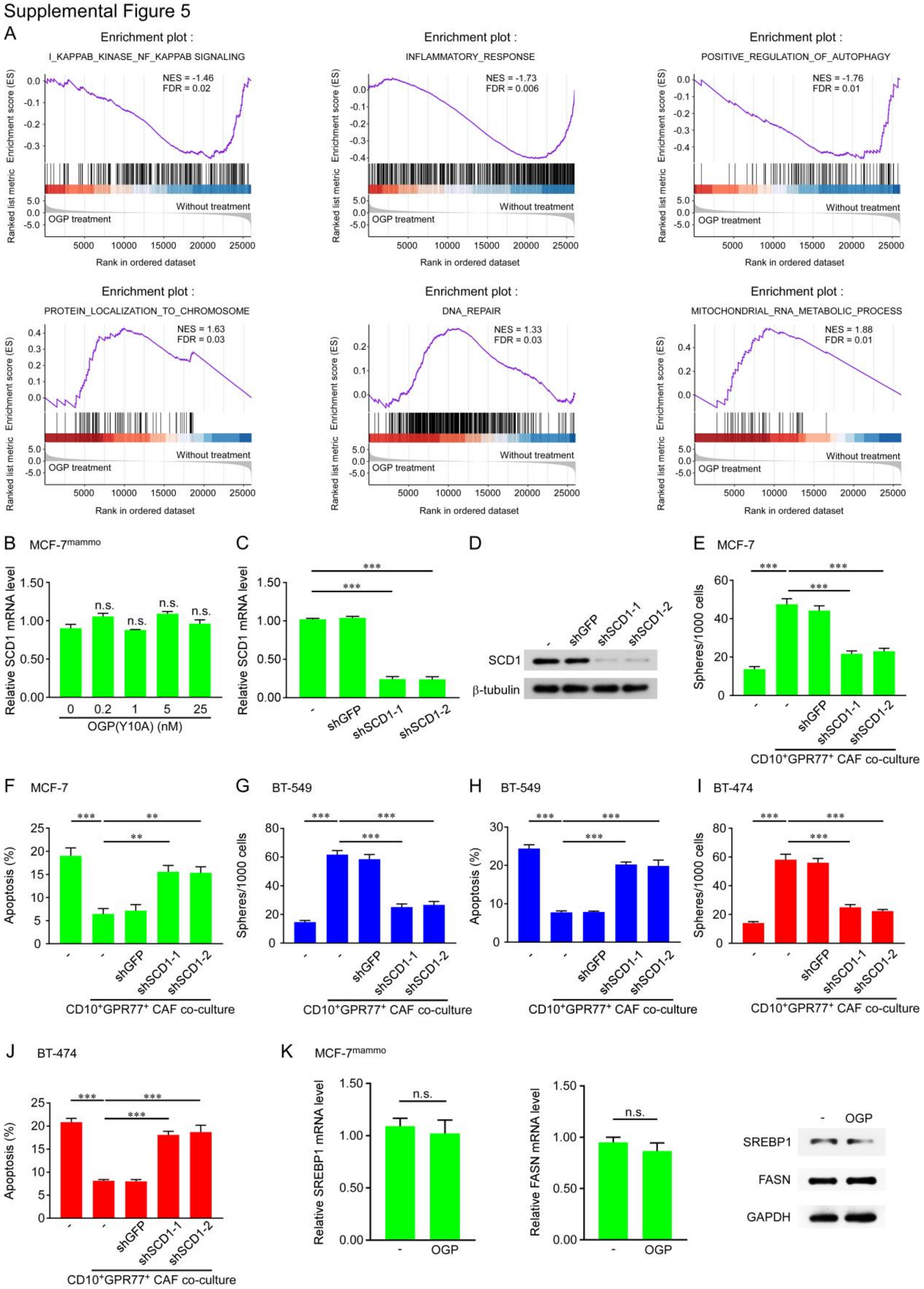

Figure S5. OGP suppresses CSCs by inhibiting lipid desaturation

A The top differentially expressed pathways in MCF-7^{mammo} cells treated with OGP were identified by the GSEA-based analysis.

B The expression of SCD1 in MCF-7^{mammo} cells treated with OGP(Y10A) at the indicated concentrations was determined by qRT-PCR. Mean ± SEM, n = 3. n.s., not significant by one-way ANOVA.

C, D MCF-7 cells were transduced with GFP shRNA or SCD1 shRNA.

C SCD1 expression was detected by qRT-PCR (n = 3).

D SCD1 levels were determined by western blotting (n = 3).

E Quantification of Figure 6K (n = 6).

F Quantification of Figure 6L (n = 3).

G-J BT-549 and BT-474 cells transduced with SCD1 shRNA were co-cultured with $CD10^{+}GPR77^{+}$ CAFs.

G, I Quantification of mammosphere formation in BT-549 **(E)** and BT-474 **(G)** cells (n = 6 for each cell line).

H, J Quantification of docetaxel-induced apoptosis of BT-549 **(F)** and BT-474 **(H)** cells determined by flow cytometry (n = 3 for each cell line).

K The expression of SREBP1 and FASN in MCF-7^{mammo} cells treated with or without OGP was detected by qRT-PCR (left) and western blotting (right). Mean ± SEM, n = 3. n.s., not significant by the Student's t-test.

C, E-J, Mean ± SEM, $^{**}P < 0.01$; $^{***}P < 0.001$ by one-way ANOVA.

Supplemental Figure 6

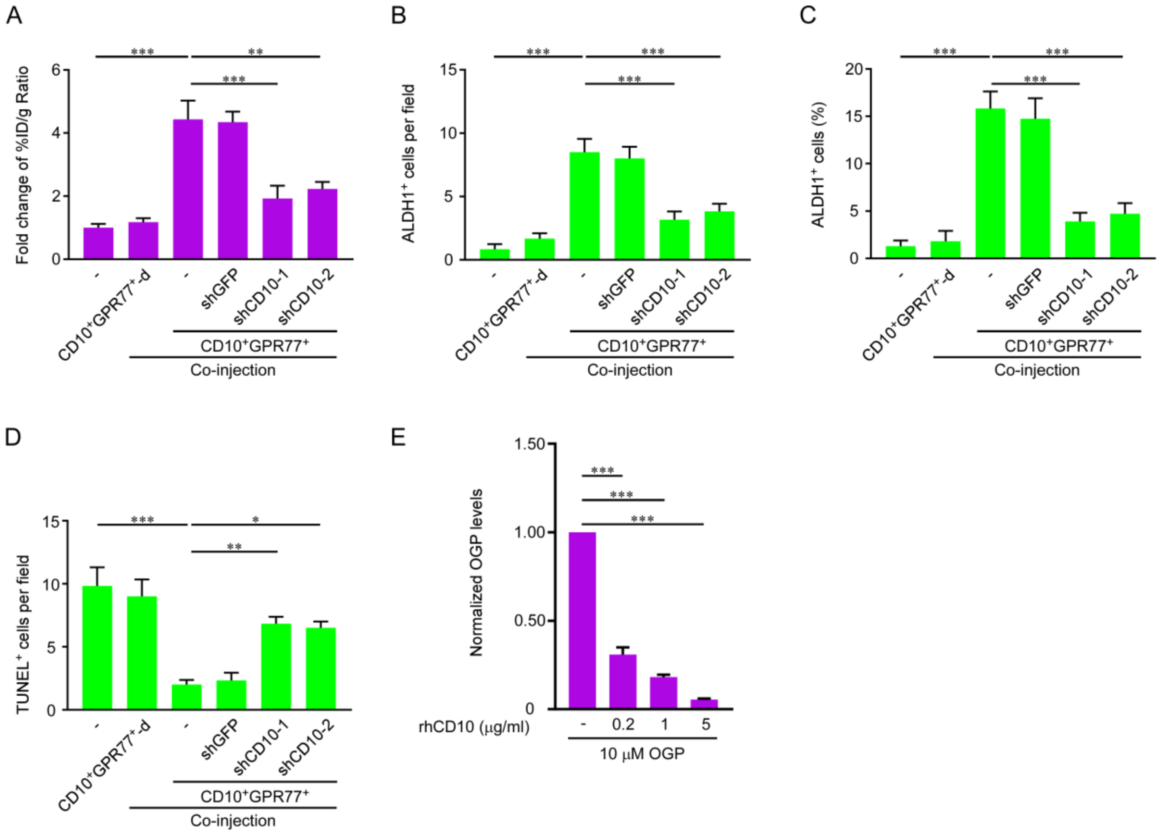

Figure S6. Hydrolyzation of OGP by CD10 provides a potential therapeutic target for cancer treatments

A Quantification of Figure 8A (n = 6 per group).

B Quantification of Figure 8C (n = 6 per group).

C Quantification of Figure 8D (n = 5 per group).

D Quantification of Figure 8E (n = 6 per group).

E OGP (10 μM) was incubated with or without the indicated concentrations of rhCD10 in 1 ml 50 mM Tris-HCl buffer (pH 7.5) at 37°C. After incubation for 1 h, the level of OGP was quantified by ELISA (n = 3).

Mean ± SEM, **P* < 0.05; ***P* < 0.01; ****P* < 0.001 by one-way ANOVA.

Reference

[1] I. Bab, D. Gazit, M. Chorev, A. Muhlrad, A. Shteyer, Z. Greenberg, M. Namdar, A. Kahn, *EMBO J.* **1992**, *11*, 1867.
